# Considering reefscape configuration and composition in biophysical models advance seascape genetics

**DOI:** 10.1371/journal.pone.0178239

**Published:** 2017-05-25

**Authors:** Simon Van Wynsberge, Serge Andréfouët, Nabila Gaertner-Mazouni, Josina Tiavouane, Daphné Grulois, Jérôme Lefèvre, Malin L. Pinsky, Cécile Fauvelot

**Affiliations:** 1 UMR-241 EIO, Université de la Polynésie Française, Laboratoire d’Excellence CORAIL, Faa’a, Tahiti, French Polynesia; 2 UMR-9220 ENTROPIE (Institut de Recherche pour le Développement, Université de La Réunion, CNRS), Laboratoire d’excellence-CORAIL, centre IRD de Nouméa, Nouméa, New Caledonia; 3 UMR-065 LEGOS and UMR-235 MIO, Centre IRD de Nouméa, Nouméa, New Caledonia; 4 Department of Ecology, Evolution, and Natural Resources, Rutgers University, New Brunswick, New Jersey, United States of America; 5 Institute of Earth, Ocean, and Atmospheric Sciences, Rutgers University, New Brunswick, New Jersey, United States of America; Universita degli Studi della Tuscia, ITALY

## Abstract

Previous seascape genetics studies have emphasized the role of ocean currents and geographic distances to explain the genetic structure of marine species, but the role of benthic habitat has been more rarely considered. Here, we compared the population genetic structure observed in West Pacific giant clam populations against model simulations that accounted habitat composition and configuration, geographical distance, and oceanic currents. Dispersal determined by geographical distance provided a modelled genetic structure in better agreement with the observations than dispersal by oceanic currents, possibly due to insufficient spatial resolution of available oceanographic and coastal circulation models. Considering both habitat composition and configuration significantly improved the match between simulated and observed genetic structures. This study emphasizes the importance of a reefscape genetics approach to population ecology, evolution and conservation in the sea.

## Introduction

The dispersal of individuals between populations is the foundation of meta-population dynamics [1]. In a meta-population context, dispersal can affect population growth and vital rates, a process termed “demographic connectivity” (Table 1) [2]. Characterizing the patterns of demographic connectivity and identifying their drivers is necessary for designing effective management plans [3, 4]. In practice however, measuring dispersal for many species at scales relevant to management questions is a difficult task [5]. Demographic connectivity is therefore often estimated with models but rarely validated against empirical field data [4].

**Table 1:** Definitions for the main technical terms used in this study, with references.

Term	Definition	References
Demographic connectivity	Process by which dispersal of propagules affects population growth and vital rates	[2]
Genetic connectivity	Degree to which gene flow affects evolutionary processes within populations	[2]
Landscape genetics	Field of study that explicitly quantifies the effects of landscape composition and landscape configuration on gene flow and spatial genetic variation	[6]
Landscape composition	Nature and relative proportion of habitat patches that shape the landscape	[7]
Landscape configuration	Habitat fragmentation and spatial arrangement of habitat patches relative to each other	[7]
Isolation by distance (IBD)	Process by which geographically restricted gene flow and random genetic drift generate genetic differentiation of populations on different habitat patches, resulting in a pattern of increasing genetic differentiation as a function of distance	[8]
Multi-disciplinary seascape genetics	Modelling approach that integrates biophysical information to adequately explain the observed population genetic structure in the marine realm	[9]
Isolation By Oceanographic Distance (IBOD)	Process by which genetic differentiation among populations is induced by the direction and intensity of water currents	[10]
Habitat patchiness	Level of fragmentation of habitat patches, from highly fragmented to continuous.	[11]
Seascape (as currently perceived in seascape genetics)	Complex mosaic of pelagic habitat patches. Pelagic habitat refers to environmental features of the water column.	-
Reefscape	Complex mosaic of benthic habitat patches in coral reef ecosystems.	-
Reefscape genetics	Field of study that explicitly quantifies the effects of reefscape composition and reefscape configuration on gene flow and spatial genetic variation. While seascape genetics mostly focused on the pelagic habitat, reefscape genetics emphasis the role of benthic habitats on genetic structure.	This study

Recent advances in population genetics and the identification of hypervariable genetic markers provide new opportunities to infer demographic connectivity by estimating gene flow among populations from allele frequencies [2]. Indeed, it is often assumed that processes driving dispersal and demographic connectivity also shape genetic patterns [12]. Genetic connectivity is the degree to which gene flow affects evolutionary processes within populations [2]. However, genetic connectivity integrates dispersal over several generations, as well as other evolutionary forces (i.e., genetic drift, selection and mutation).

Trying to relate genetic connectivity with environmental factors expected to influence dispersal has been the focus of several recent studies. This approach was termed “landscape genetics” in terrestrial ecosystems, by a fusion of “landscape ecology” with “population genetics” [13]. Landscape genetics explicitly aims to quantify the effects of landscape composition and landscape configuration on gene flow and spatial genetic variation [6]. In the landscape ecology literature, the “landscape” is conceptualized as a complex of habitat patches. The term “landscape composition” refers to the nature and relative proportion of habitat patches in the landscape, while “landscape configuration” refers to the spatial arrangement of habitat patches relative to each other [7].

The simplest form of landscape genetics relates genetic distances to geographical distances (i.e., Euclidean distance), a model termed “isolation by distance” (IBD). Isolation by distance is the process by which geographically restricted gene flow and random genetic drift generate genetic differentiation among populations on different habitat patches [8]. In terms of meta-population dynamics, IBD suggests that the exchange of individuals between habitat patches decreases as the geographical distance between them increases. Besides geographical distance, other environmental features related to landscape composition and configuration have also been used to explain genetic patterns, including habitat fragmentation, topography, snow depth, and presence/absence of rivers [14]. In these cases, unfavourable environmental features between habitat patches become barriers to gene flow, following an Isolation by Barrier (IBB) model.

The translation of landscape genetics to the marine realm was termed “seascape genetics” [9, 15], but has become its own fairly distinct field due to inherent differences of the marine environment and marine organisms. In the marine realm, dispersal of most of organisms occurs during the pelagic stage of their life cycle (e.g., as larvae), so that population genetic structure is likely related to the transport pathways of propagules [16]. In this case, the dispersal kernel is influenced both by biological and physical processes affecting the pelagic larvae [4]. Approaches that integrate biophysical information on ocean current and larval dispersal have therefore become popular [9]. These “multi-disciplinary seascape genetics” approaches [9] introduced the concept of “Isolation By Oceanographic Distance” (IBOD), which relates empirical genetic structure with a metric of oceanographic distance as the seascape feature [10]. Information integrated into the distance metric often includes the Pelagic Larval Duration (PLD) and the direction and velocity of oceanographic currents [17, 18], though some studies have also investigated the influence of other environmental features of the water column that may act as barriers to the dispersal of propagules, including upwelling, gradients in water temperature, and salinity [16].

More recently, however, several modelling and demographic studies have suggested that seascape features beyond oceanographic distance may influence demographic connectivity. This includes, for example, the spatial configuration of favourable habitat for mature adults of benthic species (i.e., benthic habitat). In coral reef ecosystems, the spatial configuration of benthic habitat is referred to as the “reefscape configuration.” Indeed, habitat patchiness may drive the relative influence of local and non-local offspring, with higher self-recruitment expected in fragmented habitats compared to continuous habitat patches [11]. At time scales of several generations, habitat patchiness can also influence population genetic structure because distant habitat patches (which may not be connected directly by a single dispersal event) can nevertheless be indirectly connected by gene flow through intermediate habitat patches that act as stepping-stones. Stepping stone models therefore predict higher genetic structure in fragmented habitats than in continuous habitats [17]. In addition to habitat patchiness and reefscape configuration, reefscape composition is also likely to play a role in demographic connectivity. Habitat patches have to be of sufficient quality and suitability for a species to settle, survive, feed, grow and reproduce [19].

Despite these recent conceptual advances, few marine studies have tried to relate genetic structure to the spatial configuration and composition of adult habitats [20]. Improving our understanding of these links is a question of broad interest for ecology because spatial features related to reefscape may significantly influence population connectivity and functioning. In addition, a better understanding of reefscape influences on connectivity could help provide practical information for management and conservation where habitat mapping are available but larvae transport pathways are not.

**Fig 1:**
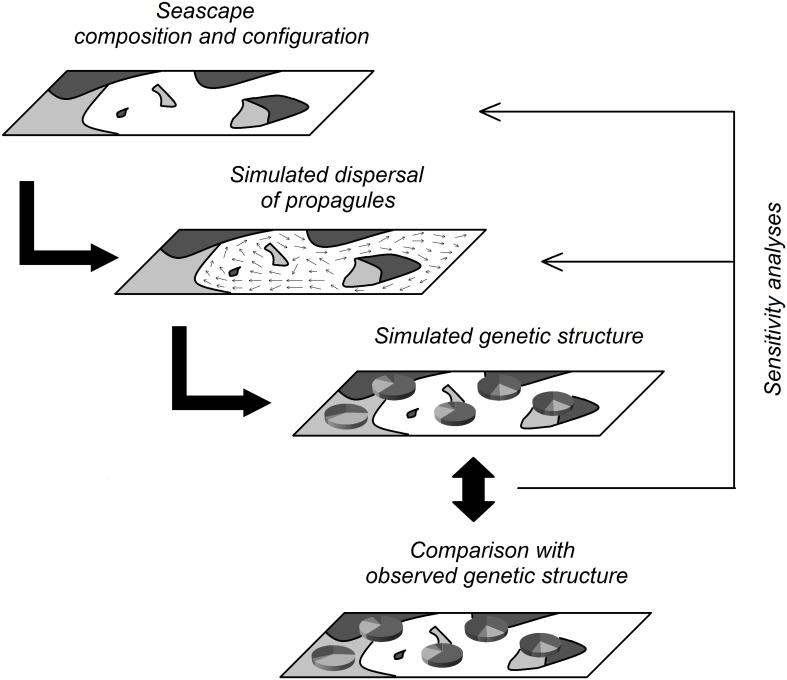
Modelling approach to explicitly test the influence of seascape composition and configuration on genetic structure. The relative influence of the various input parameters (e.g., habitat distribution and type, dispersal distance or pelagic larval duration) on genetic connectivity can be assessed quantitatively by performing sensitivity analyses of these parameters on the match between observed and simulated genetic divergences.

The hypothesis to test in this study is that reefscape configuration and composition shapes genetic patterns. We propose explicitly quantifying the influence of reefscape configuration and composition on genetic structure with a multi-disciplinary modelling approach (Fig 1) that includes (i) establishing maps of suitable habitat for the focal species; (ii) estimating the relative probability of propagule dispersal between all habitat patches based on simple Euclidian distance or complex biophysical modelling; (iii) simulating gene flow over time to account for stepping stone processes as well as other evolutionary forces (e.g., genetic drift and mutation) and to translate the relative probability of propagule dispersal into genetic distances between all pairs of habitat patches; (iv) quantitatively comparing the simulated genetic structure with the observed genetic structure; (v) and performing sensitivity analyses to evaluate the extent to which the fit between simulated and observed genetic structure is affected when the original patterns of reefscape composition and configuration are modified or degraded.

Developing such an approach that integrates habitat suitability maps, biophysical modelling, gene flow modelling and empirical genetic data is, however, complicated by the range of spatial and temporal scales inherent to these processes [21]. To date, this has only been partially achieved in the seascape literature. D’Aloia et al. [22] investigated a method to infer the dispersal kernel of the Caribbean fish *Elacatinus lori* on the Mesoamerican Barrier Reef from genetic parental data while considering the spatial distribution of habitat patches. For the same species and in the same area, D’Aloia et al. [23] tested the influence of geographical distance (IBD) and oceanic breaks (IBB) on genetic structure and found that a 20 km break in the reef could induce genetic structure. Alberto et al. [24] modelled the spatial genetic structure of the giant kelp *Macrocystis pyrifera* obtained from microsatellite empirical data, as a function of geographic distance and habitat continuity (i.e. similar to habitat fragmentation). They found a significant influence of habitat continuity on genetic distance. Nanninga et al. [25] highlighted the effect of habitat quality on population genetic patterns of an anemonefish in the Red Sea, but their study considered habitat as the type of water masses surrounding the reefs and do not consider the benthic habitat of the targeted clown fish. Other se studies integrated biophysical modelling in their analyses to test for IBOD: Davies et al. [18] tested both IBD and IBOD to explain the genetic structure and diversity of two corals (*Acropora hyacinthus* and *A*. *digitifera*) in Micronesia. They found a strong influence of geographic distance and oceanographic distance on genetic patterns and concluded that Micronesia may serve as a stepping stone pathway between the Coral Triangle in Asia (Philippines and Indonesia in particular) and the Central Pacific islands. Johansson et al. [26] investigated if oceanographic transport and other seascape features explained different scales of genetic structure of giant kelp, *Macrocystis pyrifera*. They found a significant effect of habitat continuity on genetic distance, but we are not aware of equivalent studies in coral reef ecosystems. Kool et al. [27, 28] used a matrix analysis in conjunction with a bio-oceanographic larval dispersal model to project the expected development of genetic structure in Caribbean and Indo-West Pacific coral reef ecosystems, but did not validate their model with empirical genetic data. Galindo et al. [29] used a coupled oceanographic-genetic model to predict population structure of Caribbean corals, and found a general concordance between the observed (from empirical data) and simulated genetic structure. They further highlighted that projecting connectivity forward in time provides a framework for studying long-term source-sink dynamics, making it possible to evaluate how dispersal can shape population genetic structure at regional scales [27, 28, 29]. While taking habitat maps as baseline for the oceanographic model, however, these authors did not explicitly test nor quantify the influence of reefscape composition and configuration on gene flow.

In this study, we followed the multi-disciplinary seascape modelling approach proposed above (see Fig 1) to test the hypothesis that reefscape configuration and composition shapes the population genetic structure of a common giant clam (*Tridacna maxima*) in New Caledonia and Vanuatu, South-West Pacific. The originality of our approach is about using a quantified, spatially explicit view of the benthic reef habitat of our targeted species coupled with the biophysical model. Specifically, we simulated the expected genetic divergences among giant clam populations across the study area given the observed reefscape composition and configuration and under various scenarios of larval dispersal. These simulated genetic structures were compared to the genetic structure observed from empirical genetic data. Then, sensitivity analyses explicitly quantified the importance of habitat patchiness and habitat suitability on genetic structure. The giant clam *T*. *maxima* is our model species, but our results remain relevant for a wide range of species of similar life history traits (pre-competency period around 9 days and shallow reef habitat).

## Methods

Permits for mantle biopsies of giant clams were from Direction de l'environnement de la Province Sud (n° 3117-2011/ARR/DENV, n° 2432-2012/ARR/DENV, n° 2660-2013/ARR/DENV), Direction du Développement économique et de l'environnement, Province Nord (n° 60912-25-28-2012/JJC, n° 60455-15-25/JJC), and Direction du développement économique de la Province des Iles Loyauté (n° 6161-37/PR). No permissions were required for Vanuatu.

### Study area

**Fig 2:**
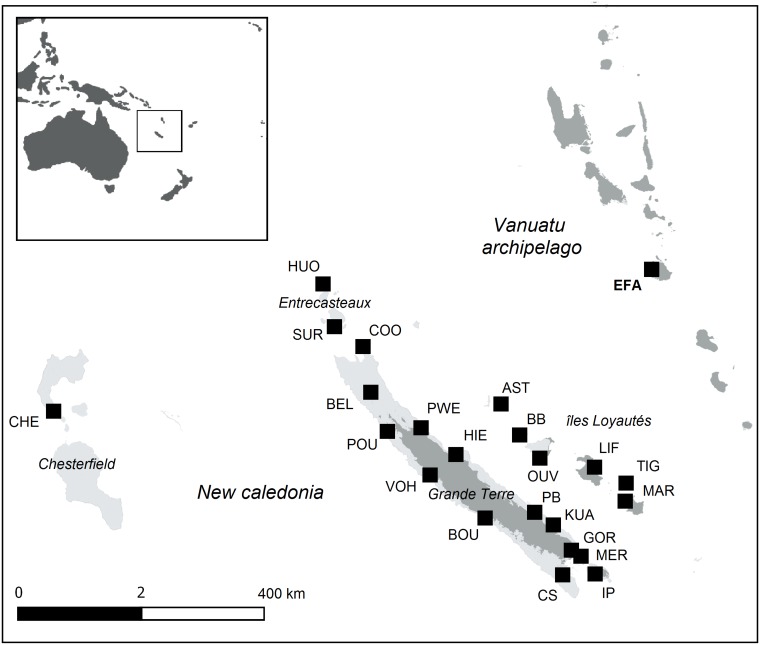
Location of New Caledonia and Vanuatu in the Pacific Ocean and of the 23 sites sampled for genetic analysis. The area extends from Efate (EFA, Vanuatu) in the East to the Chesterfield islands (CHE) in the West. Location names and sampling effort per location is available in Table 2. Land and reef maps are from the Millennium Coral Reef Mapping Project [31]. Dark grey stands for land and light grey for coral reefs.

The study area extended from 158°E to 171°E and from 24°S to 12°S. It covered Vanuatu’s oceanic islands and New Caledonia (Fig 2). Reefs of Vanuatu oceanic islands are mostly narrow fringing reefs with small, narrow lagoons. By contrast, a 1,600 km-long barrier reef surrounds both New Caledonia’s continental main island (Grande Terre) and its wide and deep 16,800 km^2^ lagoon. Around Grande-Terre, several satellite reef systems of various sizes are found. The most prominent is further west: the “Chesterfield” archipelago includes a vast and open lagoon (12,200 km^2^) with several highly exposed intertidal reefs and islands [30]. The Loyalty Islands, Entrecasteaux atolls and Ile des Pins reef systems are found respectively east, north and south of Grande Terre.

**Table 2:** Location names and number of specimen collected (n) per location for genetic analyses.

Location code	Location name	n
EFA	Efate	14
CHE	Chesterfield	19
HUO	Huon	38
SUR	Surprise	43
COO	Récif Cook	27
BEL	Belep	24
AST	Astrolabe	45
POU	Poum	45
PWE	Pouebo	45
BB	Beautemps-Beauprès	45
HIE	Hienghène	43
OUV	Ouvea	48
LIF	Lifou	46
VOH	Voh	47
TIG	Tiga	25
MAR	Mare	47
PB	Port-Bouquet	38
BOU	Bourail	46
KUA	Kuake	11
GOR	Goro	22
MER	Merlet	41
IP	Ile des Pins	42
CS	Corne Sud	41

The main features and variability in regional circulation are reviewed and described in Cravatte et al. [32]. The westward South Equatorial Current (SEC) splits into several branches when crossing Vanuatu and New Caledonia, bifurcating either northwestward via the North Vanuatu Jet and the North Caledonian Jet, or southwestward via the South Caledonian Jet. Around the southwest area of New Caledonia, the surface flow is eastward via the Sub-Tropical Counter Current and the Alis Current of New Caledonia (ACNC), with high variability due to intermittent eddies. The Loyalty Islands are separated from the Grande Terre by the Vauban Current, usually oriented southeastward, but with high intra-seasonal variability. Indeed, transport pathways in the vicinity of Vanuatu and New Caledonia archipelagos are highly complicated as a result of offshore eddies migrating westward and local transient eddies and dipole circulations formed by interactions between bottom topography and ocean dynamics [32, 33].

### Reefscape composition and configuration

**Fig 3:**
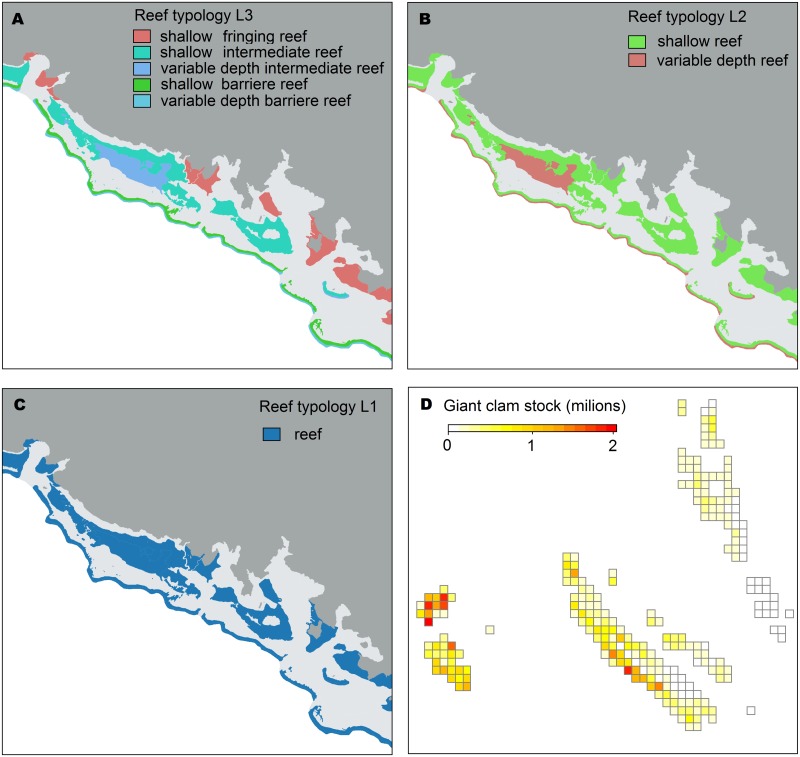
Illustration of how habitat composition and configuration were taken into account in this study. (A), (B), and (C) are habitat maps for south BOU (see Fig 2) established according to reef typology levels L3, L2, and L1, respectively (see Table 3). (D) Estimated abundance of *T*. *maxima* for each habitat patch. Abundance is the product of habitat surface and density per habitat (the figure displayed here was established on the basis of the L3 level, Table 3). Note that lower abundances were predicted in the south east part of New Caledonia and Vanuatu.

Habitat suitability maps were developed from field data on *T*. *maxima* densities and from 30 m Landsat satellite imagery from the Millennium Mapping Project [31]. Data on *T*. *maxima* densities came from Gilbert et al. [34], Wantiez et al. [35], and from Friedman et al. [36]. To avoid biases associated with combining data across multiple methods, we only considered studies that used belt transects. Overall, density estimates were available for 31 sites spread over the area. Each site was sampled with a number of stations spread over various shallow reef geomorphological units, and each station is a set of transects. Reef geomorphological units were defined at three levels (L1, L2, L3, Table 3 and Fig 3a and 3c). The geomorphological level L1 considered all reefs (shallow and variable depth reef areas confounded) as habitat for *T*. *maxima*. By contrast, level L2 distinguished shallow reefs from variable depth reefs, the latter being characterized by hundred-fold lower densities of *T*. *maxima*. Since no quantitative information on giant clam density on variable depth reefs was available in the literature, we arbitrary fixed *T*. *maxima* density on variable depth reefs at the density reported in shallow reefs divided by one hundred. Level L3 dissociated “Outer barrier reef of continental islands” (found in New Caledonia) from “Intermediate reef of continental islands” (New Caledonia), “Fringing reef of continental islands” (New Caledonia), “Oceanic patch reef of continental islands” (New Caledonia), “Oceanic islands” (Vanuatu, Lifou, Mare and Tiga), and “Atolls/banks” (Entrecasteaux, Chesterfield). Densities of all L3 categories falling in the “Variable depth reefs” L2 category were fixed at the densities of the corresponding L3 category falling in the “Shallow reed” L2 category, divided by 100. For example, the density of giant clams in Shallow Fringing reef of continental island was estimated as 256 ± 272 ind.ha^-1^ from field data, so we calculated the density of giant clams in Variable depth Fringing reef of continental island to be 2.56 ± 2.72 ind.ha^-1^ (Table 3).

**Table 3:** Typologies of reef geomorphology considered in this study.

L1	L2	L3	*Tridacna maxima* density (ind.ha^-1^)	n	References
Reef			*224* ± *193*^*a*^	*31*	[34,35,36]
	Shallow reefs		*224* ± *193* ^*a*^	*31*	[34,35,36]
		Fringing reef of continental island	*256* ± *272* ^*a*^	*3*	[34]
		Intermediate reef of continental island	*200* ± *84* ^*a*^	*9*	[34]
		Outer barrier reef of continental island	*329* ± *269* ^*a*^	*10*	[34]
		Oceanic patch reef of continental island	*104* ± *42* ^*a*^	*3*	[34]
		Oceanic island	*109* ± *93* ^*a*^	*5*	[36]
		Atoll/Bank reef	*234* ± *0* ^*a*^	*1*	[35]
	Variable depth reef		*2*.*24* ± *1*.*93* ^*b*^	-	-
		Fringing reef of continental island	*2*.*56* ± *2*.*72* ^*b*^	-	-
		Intermediate reef of continental island	*2*.*00* ± *0*.*84* ^*b*^	-	-
		Outer barrier reef of continental island	*3*.*29* ± *2*.*69* ^*b*^	-	-
		Oceanic patch reef of continental island	*1*.*04* ± *0*.*42* ^*b*^	-	-
		Oceanic island	*1*.*09* ± *0*.*93* ^*b*^	-	-
		Atoll/Bank reef	*2*.*34* ± *0*.*00* ^*b*^	-	-

The typology involves three levels: L1, L2 and L3 (see Fig 3).

For each L3 category of “Shallow Reefs” (L2 category) (marked by “*a*” in the table), *T*. *maxima* density estimates were directly derived from field surveys (the number of sampling sites “*n”* and references are provided).

The densities for L3 categories falling in the “variable depth reefs” L2 category (marked by “*b*” in the table) were systematically considered a hundred fold lower than for shallow reefs.

The continuous map of habitat was gridded at 0.25×0.25 degrees. Abundances of giant clam within each cell were calculated by multiplying estimated densities per habitat type by habitat area. The final grid was therefore a mosaic of habitat patches with different giant clam abundances (seascape composition) and different spatial relations to each other (seascape configuration) (Fig 3D).

### Dispersal kernel and connectivity matrices

Two types of dispersal kernels where considered in this study. First, we tested the IBD model by considering the relative probability for larvae to travel from habitat patch *i* to habitat patch *j* (*P*_*ij*_) as a decreasing function of geographic distance *d* (Eq 1):

$$P_{ij}=a^{d}$$ (1)

A large panel of values was tested for *a* (from 0.40 to 0.99 by steps of 0.01), providing 60 scenarios of IBD. Geographical distance between habitat patches was defined as the shortest path to reach one cell from another without crossing land. Land maps for New Caledonia, Vanuatu, and Chesterfield were rasterized (0.1 degree resolution) using the rasterize function of the raster package in R 3.1.0, and the shortest path between each pair of cells was determined using the costDistance function of the gdistance package.

Second, we tested the IBOD model by considering the probability for larvae to travel from habitat patch *i* to habitat patch *j* as a function of oceanic currents. Larval dispersal between habitat patches was simulated with Lagrangian particle tracking models. We modelled the drift of larvae for 9 days, as that is the typical time for *T*. *maxima*’s Larval Precompetency Duration (LPD) [37, 38]. During the competency period (after 9 days), we allowed larvae to settle as soon as they crossed a patch of favourable habitat [39, 40]. We considered a competency period ranging from 9 to 19 days on the basis of Jameson [37] and Neo et al. [40], with the number of surviving larvae exponentially decreasing with time. We used a survival curve similar to Wood et al. [41] (See S1 Fig).

The drift of particles through oceanic waters was simulated using the Roms OFfline Floats (Roff, [42]) model based on a Regional Ocean Modeling System (ROMS, [43], see S1 File for details) with a 1/12° (about 8 km) horizontal resolution that adequately reproduces mesoscale details of the mean flow [33]. Since no seasonality in recruitment is evident for *T*. *maxima* in New Caledonia, the rate of particle release was set to one per day from each cell from the top depth layer over the period 1993 to 2010. This was a period long enough to capture the main features and variability of the regional circulation, as well as unusual years. Overall, 15×10^5^ particles were released (6,500 per cell), which is expected to produce robust estimations of dispersal [44]. For each simulation, we identified the habitat patches where larvae were expected to settle considering oceanographic currents. This provided a dispersal matrix *D*_*ij*_ containing the probability of transport for larvae between patches *i* and *j* through oceanic waters.

### Modelling gene flow

To evaluate the expected genetic structure across the study area under a given hypothesis of larval dispersal, we modelled gene flow among populations with a matrix projection model following Kool et al. [27]. For a given locus and allele, we defined Q_t_ as a 243 row matrix containing allele frequency among the 243 habitat patches (Eq 2). Model simulations started with an initial matrix *Q*_*0*_ that contained frequency of alleles randomly (99 simulations) distributed among habitat patches.

$$Q_{t}=\left[\begin{array}{c} F_{1}\\ \vdots \\ F_{243} \end{array}\right]$$ (2)

The model then projected the allele frequency over time according to Eq (3):

$$Q_{t+1}=\bar{AQ_{t}+NQ_{t}}$$ (3)

where N is the number of individuals in each habitat patch, calculated from patch area and *T*. *maxima* density per habitat. The density per habitat was randomly generated from a Gaussian distribution with mean and standard deviation estimated from our literature review (Table 3). *A* is a square matrix (Eq 4) containing the number of migrants dispersing between pairs of habitat patches from *t* to *t+1* (i.e., the realized larval connectivity *sensu* Watson et al. [45]).

$$A=\;\left[\begin{array}{ccc} A_{1,1} & \ldots & A_{1,243}\\ \vdots & \ddots & \vdots \\ A_{243,1} & \ldots & A_{243,243} \end{array}\right]$$ (4)

The interpretation of Eq 3 is that the expected state of the system (*Q*) at *t+1* is the result of migration progeny (*AQ*_*t*_) added to the original parent generation (*NQ*_*t*_). As *Q* refers to a matrix of allelic frequencies, a row normalisation among alleles was performed at each time step (indicated by top bar in Eq 3) so that the values always ranged between 0 and 1.

The number of migrants transferring from habitat patch *i* to habitat patch *j (A)*_*ij*_ during a generation was considered time invariant and was calculated as the number of clams in habitat patch *i* (*N*_*i*_), multiplied by a fertility rate *f* (i.e., number of adult clams produced per adult clam during a generation) to obtain the progeny (*fN*_*i*_). The number of progeny was then multiplied by *D*_*ij*_ (see previous section) to determine their distribution among habitat patches.

Under the influence of migration (i.e., dispersal events) and in the absence of drift, the allele frequencies between pairs of sites were progressively homogenised. The aim of the model was thus to identify which pairs of sites were homogenised more quickly than others to test our hypothesis about dispersal and test the relative importance of geographic distance, oceanographic currents, giant clam abundance, and habitat patchiness.

### Comparison of simulated versus observed genetic structures

The simulated genetic structures obtained from the simulation approach described above were then compared to observed genetic structure. Mantle biopsies of 849 *T*. *maxima* spread over 23 sampled sites were conducted from 2008 to 2014 (Fig 2, Table 2) and each individual was genotyped at 15 microsatellite loci as described in Grulois et al. [46].

Genetic diversity within samples was estimated using observed (H_obs_) and Nei’s unbiased expected heterozygosity (H_exp_) in GENETIX version 4.03 [47]. Single and multilocus *F*_IS_ were estimated using Weir & Cockerham’s [48] fixation index and deviations from Hardy-Weinberg equilibrium (HWE) were tested using Fisher’s exact test using a Markov-chain randomization (1000 dememorizations, 100 batches, and 1000 iterations per batch) in GENEPOP version 3.4 [49] as implemented for online use (http://genepop.curtin.edu.au/). Significance levels for multiple comparisons of loci across samples were adjusted using a standard Bonferroni correction [50].

Since null alleles were detected in our samples, genetic divergences among samples were estimated in FREENA [51] (i) using the *F*_ST_ estimates of Weir & Cockerham [52] and the ENA method that provides unbiased *F*_ST_ estimates, and (ii) using a direct estimation of pairwise values for the Cavalli-Sforza & Edwards [53] genetic distance following the so-called INA method [51] that decreases the bias in genetic distance estimation observed when null alleles are present. Genotypic differentiation among samples was further tested with an exact test (Markov chain parameters: 1000 dememorizations, followed by 1000 batches of 1000 iterations per batch) with the original dataset, and the *P*-value of the log-likelihood (G) based on the exact test [54] was estimated in GENEPOP version 3.4. Significance levels for multiple comparisons of loci across samples were adjusted using a sequential Bonferroni correction [50].

Among the dispersal kernels tested (IBD and IBOD), we identified those most similar to the observed genetic structure by comparing the observed and simulated standardized Cavalli-Sforza & Edwards’s [53] genetic distances. The simulated genetics distances (*d*_*s*_) were compared with the observed genetic distances (*d*_*o*_), using Mantel’s correlation coefficient for matrices.

### Sensitivity analyses of the genetic structure to seascape features

Among all dispersal kernels tested in the previous sections, the one that simulated a genetic structure most similar to the observed structure was then used to evaluate the importance of reefscape composition and configuration in genetic patterns. The importance of reefscape composition was evaluated by degrading the high resolution habitat map (L3) to coarser habitat maps (down to L2 and L1). This provided three maps of habitats, each characterized by a more or less accurate spatial distribution of *T*. *maxima* density (Fig 3).

To evaluate if reefscape configuration (i.e. habitat continuity) and composition need to be taken into account for modelling genetic structure accurately, we compared the correlation between the observed and the simulated genetic structures obtained when considering the original habitat maps with the correlation obtained when degrading the original habitat map. Degradation of the original habitat map was performed by progressively reducing habitat area between populations surveyed for genetic data. For this, we respectively tested decreases from 20% to 80% of the initial habitat area. Patches that were removed were randomly chosen among patches not surveyed for genetic samples. These scenarios represented the process of habitat fragmentation that generates habitat loss and increases geographical distance between remaining habitat patches. All scenarios tested in this study are summarized in Table 4.

**Table 4:** Summary of all scenarios tested in this study and the corresponding correlations between the simulated and observed genetic structure.

Scenarios	Dispersal kernel	Habitat fragmentation	Habitat composition	Mantel coefficient of correlation
1	IBD	0	L3	0.66 ± 0.08
2	IBD	20	L3	0.58 ± 0.12
3	IBD	40	L3	0.56 ± 0.12
4	IBD	60	L3	0.53 ± 0.14
5	IBD	80	L3	0.50 ± 0.14
6	IBD	0	L2	0.58 ± 0.14
7	IBD	20	L2	0.50 ± 0.19
8	IBD	40	L2	0.45 ± 0.18
9	IBD	60	L2	0.47 ± 0.17
10	IBD	80	L2	0.47 ± 0.18
11	IBD	0	L1	0.63 ± 0.07
12	IBD	20	L1	0.51 ± 0.14
13	IBD	40	L1	0.40 ± 0.20
14	IBD	60	L1	0.41 ± 0.16
15	IBD	80	L1	0.44 ± 0.22
16	IBOD	0	L3	0.07 ± 0.09

## Results

### Giant clam observed genetic diversity and structure

Raw microsatellite data can be found at https://figshare.com/s/a00f8be352aae916c147. Over all samples, the number of alleles per locus ranged from 11 to 40 (mean 26.8). The observed heterozygosities ranged from 0.493 to 0.610, and the expected heterozygosities ranged from 0.752 to 0.831 (See S1 Table). Significant deviations from HWE were observed in all samples, with multilocus estimates of *F*_IS_ ranging from 0.273 to 0.402, showing in all cases heterozygote deficiencies. However, six loci were in HWE in nearly all samples, suggesting that heterozygote deficiencies in the remaining nine loci were caused by the occurrence of null alleles (confirmed by the presence of null homozygotes at those nine loci).

The Efate sample (Vanuatu) was significantly differentiated from other sampled sites with pairwise Cavalli-Sforza & Edwards’ genetic distance values ranging from 0.388 to 0.467 (all *P*-values < 0.01, see S2 Table). The Chesterfield sample was also divergent from New Caledonia’s and Vanuatu’s samples (Cavalli-Sforza & Edwards’ genetic distance from 0.306 to 0.412, though not significant). Lower levels of genetic differentiation were observed among the sampled sites of New Caledonia (Cavalli-Sforza & Edwards’ genetic distance values from 0.235 to 0.38), but some sites in the north of New Caledonia (BEL and COO), the Loyalty islands (TIG) and the south of New Caledonia (GOR) held higher (but not significant) genetic distances with other sites. The KUA sample was also characterized by higher genetic distances with other sites (from 0.345 to 0.401), but was possibly biased by the low number of specimen collected at this station (n = 11, Table 2). This likely biased sample was excluded from further analyses.

### Dispersal kernel, connectivity matrices, and genetic structure

**Fig 4:**
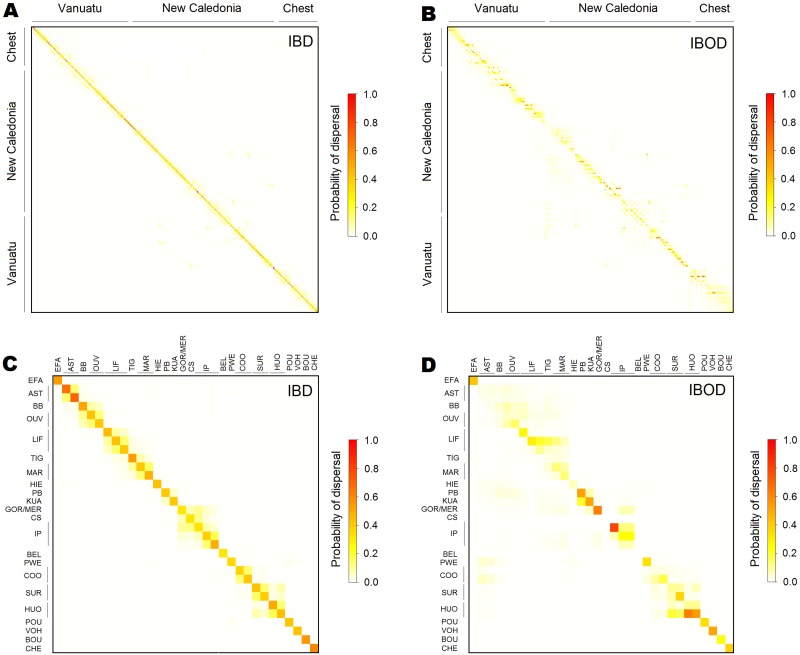
Connectivity matrices between all habitat patches considering an IBD dispersal kernel (A and C) or an IBOD dispersal kernel (B and D). A) and B) display all habitat patches (see Fig 3D), while C) and D) only display habitat patches that were sampled for genetic analyses (see Fig 2).

The bio-physical oceanographic models showed that larvae released in the northern part of Vanuatu archipelago (above 17°S) tended to be exported northwestward by the North Vanuatu Jet (NVJ) where no patch of suitable habitat exists. The occurrence of successful exports of larvae from Vanuatu to New-Caledonia progressively increased for release points further south, but successful exports were restricted to the Loyalty Islands, New Caledonia’s East coast, and Entrecasteaux reefs through the East Caledonian Current (ECC), the North Caledonian Jet (NCJ) and the South Caledonian Jet (SCJ). By contrast, a significant fraction of larvae could cross the distance between the north part of New Caledonia and the Chesterfield Islands using eddies issued from the NCJ and the Sub-Tropical Counter Current (STCC). This provided a connectivity matrix with higher connections among habitat patches of New Caledonia than between Vanuatu, New Caledonia, and Chesterfield (Fig 4B).

The dispersal kernel that provided the best match with genetic data was the IBD scenario with *a* = 0.95 in Eq 1 (S2 Fig). The connectivity matrix for this scenario is provided in Fig 4A and suggests high self-recruitment and restricted dispersal. For this scenario, dispersal was restricted to very proximate patches (e.g. between AST and BB or between LIF and TIG), and never occurred beyond 150 km (S3 Fig).

**Fig 5:**
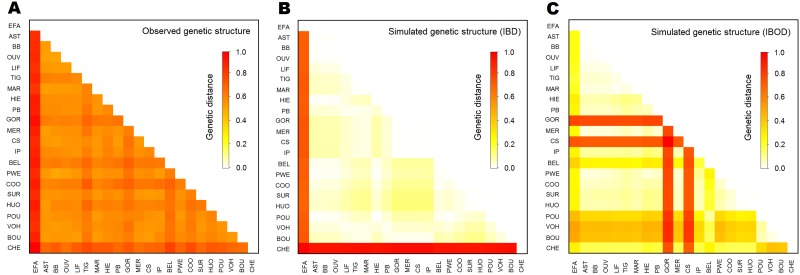
Comparison between observed and simulated genetic structures for *Tridacna maxima*. A) Observed genetic distances from empirical data, obtained from 15 microsatellite loci. B) Genetic distances simulated by the isolation by distance (IBD) model. C) Genetic distances simulated by the isolation by oceanographic distance (IBOD) model. Genetic distances are Cavalli-Sforza & Edwards’s genetic distances [53], normalized to have maximum of 1.

When considering an IBD pattern for dispersal, the gene flow model adequately isolated Vanuatu and Chesterfield from New-Caledonia, but did not adequately mimic the genetic structure among New-Caledonia’s reefs (Fig 5). By contrast, the IBOD model failed to project isolation for Chesterfield islands, and estimated higher relative isolation for the south group than was observed in the empirical genetic data (Fig 5).

### Sensitivity analyses of genetic structure to seascape features

**Fig 6:**
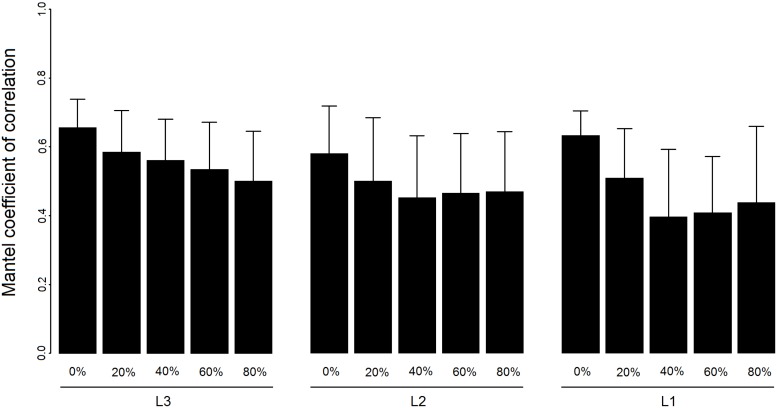
Correlations between observed and simulated genetic structures for the various scenarios of landscape composition and configuration. Landscape composition was determined by the three levels of the reef typology described in Table 2. Landscape configuration is determined by habitat fragmentation (0% refers to the initial habitat maps, while 20%, 40%, 60% and 80% refer to reduction levels of habitat area compared to the initial habitat map). The coefficient of correlation is the Mantel coefficient of matrix correlation. We used the dispersal kernel that provided the best congruence between the observed and simulated genetic distances (IBD). Bars are medians and error bars are quantile 95%.

Degrading the original habitat map by reducing habitat area significantly decreased the correlation between simulated and observed genetic distances (F = 13.8, p < 0.01; Fig 6). When only 20% of the initial habitat area was maintained (i.e. 80% fragmentation level), correlation decreased from 0.66 ± 0.08 to 0.50 ± 0.14 for the L3 scenario, from 0.58 ± 0.14 to 0.47 ± 0.18 for the L2 scenario, and from 0.63 ± 0.07 to 0.44 ± 0.22 for the L1 scenario. The influence of habitat composition was also significant (F = 11.6, p < 0.01), with correlation values lower for the “L1” and “L2” scenarios than for the “L3” scenario, especially when habitat fragmentation was 40%, 60% and 80%.

## Discussion

In this study, we simulated the expected genetic differentiations of giant clams across the study area given the reefscape composition and configuration and under various scenarios of larval dispersal. The simulated genetic structures were compared with the observed genetic structure established from empirical genetic data. This general framework is an example of the multi-disciplinary approach recommended by Selkoe et al. [12]. The modelling approach here involves (i) maps of habitat suitability of the species over the study area; (ii) biophysical modelling for propagule dispersal between all habitat patches; (iii) gene flow modelling that accounted for multi-generational stepping stone processes; and (iv) sensitivity analyses to evaluate the extent to which reefscape configuration and composition are necessary for modelling genetic structure accurately. This simulation approach led to similar conclusions than a regression approach (see S2 File), but could explicitly test the influence of environmental features on genetic patterns, while taking into account many of the complex processes that drive larval connectivity. This approach is a promising alternative to the simple regressions classically used in landscape or seascape studies, when habitat fragmentation/continuity can not be resumed by a single variable that would influence genetic patterns in a linear way, and interact with other factors (e.g., oceanography) also in a linear way.

The approach adopted here has a number of limitations that are important to acknowledge. First, except between Vanuatu and other samples, genetic differentiation levels were found to be low in the study area. Landscape/seascape genetics traditionally aims to evaluate genetic structure and relate it to environmental features. Faint genetic structure requires addressing the problem in a different way: which environmental features can explain the significant or non-significant genetic differentiations observed among sites? While lack of structure is not usually the focus of landscape geneticists [13], it is likely to be an important focus for seascape genetics because marine species usually have low levels of genetic structure at the spatial scale of concern for management purposes [55, 56, 57]. The limited (but present) genetic structure observed for giant clams in our study (see Vanuatu, S2 Table, Fig 5) is therefore representative of, and a useful example for, future studies in the seascape literature.

Second, the multi-disciplinary approach performed here required extensive datasets that were not easy to collect and bring together. Habitat maps were readily available, but a typology adapted to the current knowledge on *T*. *maxima* density in the literature needed to be established (Table 3) to represent *T*. *maxima* habitats adequately [31]. For example, comprehensive data on giant clam density in each habitat patch were not available at the wide geographical scale of our study. Specifically, we had to use the density of giant clams in each habitat type to calculate giant clam abundances for each habitat patch. We used a dataset from the literature that itself involved a substantial sampling effort (n = 31 sites surveyed over 6 shallow geomorphological units), though many regions and species do not have such data readily available. Despite the effort, we still lacked fine density estimations for reefs of variable depths (Table 3). Our results nevertheless demonstrated that more accurate characterization of reefscape features enhanced our understanding of genetic structure.

Third, the model used in this study projected gene flow on the basis of migration only (through larval dispersal). Other evolutionary forces (selection, mutation, and genetic drift) were neglected, but their influence on the results should be assessed in future research programs. Mutation is likely unimportant at the small spatial and temporal scales that are addressed in this study. Selection may affect parts of the genome including neutral genomic regions [58], but it was unlikely the case in our study because all microsatellite loci provided similar F_st_ values (S3 File). Then, genetic drift may accentuate the divergence of low density (i.e. low population size) clam habitat. Integrating processes involved in demographic connectivity and genetic connectivity is not an easy task and requires careful consideration of the life history and natural history of the species being studied. For long-lived species like giant clams, special consideration will be required in future research to handle overlapping generations in the model. Overlapping generations may modify gene flow and the relative influence of evolutionary forces on genetic structures [59].

Finally, we used the Mantel correlation coefficient to compare simulated *versus* observed genetic distances, but the Mantel test has been recently criticized due to low robustness to spatial autocorrelation [60, 61]. While using the Mantel correlation may have underestimated correlation coefficients between simulated *versus* observed genetic distances, it is unlikely to have changed the conclusions of our study which are based on a relative comparison of correlation coefficients between scenarios and not on the absolute value of correlation.

### The relatively poor performance of IBOD compared to IBD in our study

Only by considering IBD with limited dispersal (*a* = 0.95 in Eq 1) did the model project higher isolation for Vanuatu and Chesterfield, in agreement with observed genetic data (Fig 5). This result suggested that larval dispersal was restricted to short distances, usually several tens of kilometres, and rarely exceeded 150 km for giant clams in the New Caledonia and Vanuatu area (S3 Fig), a distance supported by an independent analysis which estimated the genetic patch size from a spatial auto-correlation analysis from the New Caledonia genetic survey (see S4 File). This result is also in agreement with dispersal distances estimated in other areas for species with relatively short PLD [62, 63, 64].

The IBD model projected relative isolation for Vanuatu and Chesterfield that was similar to the observed genetic divergence (Fig 5), but this was not the case for IBOD. Conversely, none of the tested scenarios could explain the genetic structure observed at finer scales, such as the higher (but not significant) isolation found for some sites in the north of New Caledonia (BEL and COO), the Loyalty islands (TIG) and the south of New Caledonia (GOR). The relatively poor performance of IBOD model compared to IBD may result from the fact that we only considered ocean currents in IBOD, while neglecting connections through lagoon waters. Much of *T*. *maxima* specimen in the area are found in the lagoon of New Caledonia, and for them the current approach does not accurately model larval dispersal at the beginning and end of the dispersal period. The movements of lagoon waters are generally driven by tide, wind and swell [65] and reef configuration [66]. In New Caledonia wide and geomorphologically complex lagoon [30], current intensities and directions remain poorly characterized especially around shallow reefs, but may produce a range of possible distances for larval dispersal [67] which may shape the fine scale genetic structure over the long run.

Our biophysical model (IBOD) considered the PLD, larval survival, and ability for larvae to settle when crossing a suitable substrate. Available computer time limited our ability to run dedicated sensitivity analyses for each of these parameters, but all are expected to influence the dispersal kernel [40] and warrant further investigation. A number of other processes also deserve more attention for future modelling of larval dispersal, including *1)* the movement of gametes before fertilization, which last several to tens of hours for giant clams [40] and may significantly influence dispersal kernel; *2)* larval swimming speed, which may also affect the path of larvae and is low for *T*. *squamosa* (104.0 to 1010.6 μm.s^-1^, [40]) but remains unestimated for *T*. *maxima* (though larval behaviour experiments reveal active swimming, [39]); *3)* factors inducing settlement, including crustose coralline algae [40] or conspecifics [39]; and *4)* spatial differences in larval survival, such as from spatial variation in predation or food availability. Finally, it is worth noting that despite the recent advances in characterizing the circulation around New Caledonia [32], ROMS models continue to have difficulty simulating currents along the coastlines, particularly in the Loyalty channel where the modeled Vauban current is too strong. Accurate knowledge of eddy dynamics is also lacking for the West and South part of New Caledonia. This could bias dispersal kernels in these areas and explain the low performance of IBOD models. However, the model available for New Caledonia remains far better than what is available in most coral reef ecosystems and has been validated extensively against empirical observations (see S1 File). Since the IBOD model was not supported by genetic data, it was not used in our analysis to test the hypothesis that reefscape configuration and composition influence genetic structure. Future research needs to improve the accuracy of the oceanographic model so that a reliable evaluation of the effect of reefscape features in an IBOD model can be performed.

### Reefscape genetics: Accounting for reef distribution in seascape genetics

The idea that habitat fragmentation and composition can impact *demographic* and *genetic* connectivity patterns has strong foundations in the terrestrial literature [13, 6]. The marine realm, by contrast, has often been considered a relatively continuous environment devoid of barriers for dispersal. The concept of “*seascape genetics*” only recently emerged in marine ecology as studies began linking connectivity patterns for geographic features. Thus far, seascape genetics has mostly used meso-scale oceanic features (i.e., currents, [68]) to describe this seascape, even for species and populations that spend most of their life in coastal benthic habitats. Specifically in tropical marine ecosystems, the diversity of reef configurations induced by contrasting patterns of reef patchiness and other reefscape features (e.g., reef geomorphology, lagoon enclosure) may also shape connectivity [11]. In this study, we highlight that even relatively simple environmental features inherent from the landscape ecology literature can be important for modelling and understanding genetic patterns for a marine species. These environmental features, related to hard bottom habitat composition and configuration, can therefore enhance our understanding of genetic patterns in coral reef ecosystems. To accurately model the genetic structure of reef species in a region, we stress the need for a specific approach that integrates reef habitat composition and configuration with oceanic and lagoon currents, hence a “*reefscape genetics*” approach.

### Reefscape features as proxy for connectivity: Perspectives for future research

Population connectivity often enhances resilience to climate change and other anthropogenic impacts on ecosystems [69]. Loss of connectivity due to habitat destruction and global warming is therefore raising concerns. Connectivity has been the focus of many investigations, but to date, seascape genetic studies typically focus on larval transport (e.g., oceanographic currents) rather than production and settlement sites. Accurately modelling larval transport is often challenging and costly, however, and requires specialized computing capabilities. To date, most coral reef ecosystems lack such detailed information, which limits the ability of biophysical models to help characterize demographic and genetic connectivity in a wide variety of places. In this context, reef biologists and managers would benefit from simple and accurate predictors of gene flow. We propose that reefscape genetics and a new focus on habitat geography can offer a fresh perspective.

Seascape features can provide useful spatial surrogates for population genetic structure that are potentially relevant for a wide range of marine organisms. Our study concluded that habitat composition and configuration significantly influenced genetic patterns of giant clams. Further investigation is needed to assess the robustness of this result to species traits (e.g., PLD), but habitat maps and other reefscape features may provide a new path for marine ecology in regions where more detailed information on ocean currents are lacking.

We suggest future research to investigate the following directions. First, one important advance will be to quantify the relative influence of a wider variety of reefscape features on genetic structure. We here demonstrated that reefscape composition and configuration can enhance our understanding of population connectivity through a reduction of habitat area and a degradation of habitat map, but the influence of reefscape features like reef enclosure (e.g., atoll *versus* island reef) should also be investigated. It would also be helpful to compare results from reefscape genetics among taxa and identify proxies of connectivity robust enough to be related to particular species traits, including PLD. Over the long run, this could help elevate reefscape genetics from a population to community scale and help characterize the link between reefscape features and biodiversity broadly. Finally, it will be useful to characterize how reef use, reef degradation, and other aspects of environmental change affect genetic connectivity, and to assess the consequences for reef management and for the identification of priority areas for conservation. These three recommendations are not exhaustive, but would help reinforce reefscape genetics as a new path for marine ecology and conservation science.

## Supporting information

S1 Table Summary of genetic diversity at 15 microsatellite loci from *Tridacna maxima* samples. n: number of sampled individuals; *H*_obs_: observed heterozygosity; *H*_exp_: Nei 's unbiased expected heterozygosity; *F*_IS_: Weir and Cockerham’s (1984) estimate of Wright’s (1951) fixation index (italic type indicate significant deviations from HWE after standard Bonferroni correction). Full names of abbreviated sampled locations are given in Fig 2.
(PDF)

S2 Table Observed pairwise genetic differentiation among *T*. *maxima* sampled locations. Above diagonal: pairwise *F*_ST_; below diagonal: Cavalli-Sforza and Edwards (1967) genetic distance used for all comparisons with simulated genetic structures. Significant genotypic differentiations are indicated in italic for *P*-value < 0.01 and in bold for significant after sequential Bonferroni correction. Full names of abbreviated sampled locations are given in Fig 2.
(PDF)

S1 Fig Relative probability of survival for *Tridacna maxima* larvae over the competency period. Competency period considered is from 9 to 19 days, and the relative probability of survival was considered for calculation of dispersal probability in the IBOD model.
(PDF)

S2 Fig Performance of IBD models to reproduce the observed genetic structure as a function of parameter a (see Eq 1). A) Results obtained when using the mantel coefficient of matrix correlation. B) Results obtained when using the linear R-squared. Points are medians and error bars are quantile 5% and 95%.
(PDF)

S3 Fig IBD dispersal kernel that provided best congruence between the simulated and observed genetic structures for *T*. *maxima* in the New Caledonia and Vanuatu area. (PDF)

S1 File Background surface circulation and model configuration. (PDF)

S2 File Linear regression models between genetic distance, geographic distance, oceanographic distance, and habitat continuity. (PDF)

S3 File F_st_ values per locus obtained with and without using ENA. (PDF)

S4 File Indirect estimates of gene dispersal distance from empirical genetic data using Moran’s *I* relationship coefficients. (PDF)

## References

[1] Hanski I. Metapopulation dynamics. Nature 1998;396: 41–49.

[2] Lowe WH, Allendorf FW. What can genetics tell us about population connectivity? Mol. Ecol. 2010;19: 3038–3051. 10.1111/j.1365-294X.2010.04688.x 20618697

[3] Fogarty MJ, Botsford LW. Population connectivity and spatial management of marine fisheries. Oceanography 2007;20: 112–123.

[4] Cowen RK, Sponaugle S. Larval Dispersal and Marine Population Connectivity. Annu. Rev. Mar. Sci. 2009;1: 443–466. 10.1146/annurev.marine.010908.163757 21141044

[5] Von der Heyden S, Beger M, Toonen RJ, van Herwerden L, Juinio-Menez MA, Ravago-Gotanco R, et al The application of genetics to marine management and conservation: examples from the Indo-Pacific. Bull. Mar. Sci. 2014;90: 123–158.

[6] Storfer A, Murphy MA, Evans JS, Goldberg CS, Robinson S, Spear SF, et al Putting the ‘landscape’ in landscape genetics. Heredity 2007;98: 128–142. 10.1038/sj.hdy.6800917 17080024

[7] Fahrig L, Nuttle WK. Population ecology in spatially heterogeneous environments In: Weathers KC editor. Ecosystem function in heterogeneous landscapes. New York: Springer; 2005 pp. 95–118.

[8] Wright S. Isolation by distance under diverse systems of mating. Genetics 1946;31: 39–59. 21009706 10.1093/genetics/31.1.39 PMC1209315

[9] Selkoe KA, Henzler CM, Gaines SD. Seascape genetics and the spatial ecology of marine populations. Fish. Fish. 2009;9: 363–377.

[10] White C, Selkoe KA, Watson J, Siegel DA, Zacherl DC, Toonen RJ. Ocean currents help explain population genetic structure. P. R. Soc. B-Biol. Sci. 2010;282: rspb2009.2214. 10.1098/rspb.2009.2214 PMC2871860 20133354

[11] Pinsky ML, Palumbi SR, Andréfouët S, Purkis SJ. Open and closed seascapes: Where does habitat patchiness create populations with high fractions of self-recruitment? Ecol. Appl. 2012;22: 1257–1267. 22827133 10.1890/11-1240.1

[12] Sale PF, Van Lavieren H, Ablan Lagman MC, Atema J, Butler M, Fauvelot C, et al. Preserving Reef Connectivity: A Handbook for Marine Protected Area Managers. Connectivity Working Group, Coral Reef Targeted Research & Capacity Buiding for Management Program, UNU-INWEH; 2010.

[13] Manel S, Schwartz MK, Luikart G, Taberlet P. Landscape genetics: combining landscape ecology and population genetics. Trends Ecol. Evol. 2003;18: 189–197.

[14] Storfer A, Murphy MA, Spear SF, Holderegger R, Waites LP. Landscape genetics: where are we now? Molecular ecology 2010;19: 3496–3514. 10.1111/j.1365-294X.2010.04691.x 20723061

[15] Manel S, Holderegger R. Ten years of landscape genetics. Trends Ecol. Evol. 2013;28: 614–621. 10.1016/j.tree.2013.05.012 23769416

[16] Selkoe KA, Scribner KT, Galindo HM. Waterscape genetics—Applications of landscape genetics to rivers, lakes, and seas In: Balkenhol N, Cushman S, Storfer A, Waits L, editors, Landscape Genetics: Concepts, Methods, Applications. New York: John Wiley & Sons, Ltd; 2016 pp 220–246.

[17] Crandall ED, Treml EA, Barber PH. Coalescent and biophysical models of stepping-stone gene flow in neritid snails. Mol. Ecol. 2012;21: 5579–5598. 10.1111/mec.12031 23050562

[18] Davies SW, Treml EA, Kenkel CD, Matz MV. Exploring the role of Micronesian islands in the maintenance of coral genetic diversity in the Pacific Ocean. Mol. Ecol. 2015;24: 70–82. 10.1111/mec.13005 25407355

[19] Bidegain G, Bàrcena JF, Garcia A, Juanes JA. LARVAHS: Predicting clam larval dispersal and recruitment using habitat suitability-based particle tracking model. Ecol. Model. 2013;268: 78–92.

[20] Riginos C, Liggins L. Seascape genetics: Populations, individuals, and genes marooned and adrift. Geography compass 2013;7: 197–216.

[21] Liggins L, Riginos C. Taking the plunge: an introduction to undertaking seascape genetic studies and using biophysical models. Geography compass 2013;7: 173–196.

[22] D'Aloia CC, Bogdanowicz M, Majoris JE, Harrison RG, Buston PM. Self-recruitment in a Caribbean reef fish: a method for approximating dispersal kernels accounting for seascape. Mol. Ecol. 2013;22: 2563–2572. 10.1111/mec.12274 23495725

[23] D’Aloia CC, Bogdanowicz SM, Harrison RG, Buston PM. Seascape continuity plays an important role in determining patterns of spatial genetic structure in a coral reef fish. Mol. Ecol. 2014;23: 2902–2913. 10.1111/mec.12782 24803419

[24] Alberto F, Raimondi PT, Reed DC, Coelho NC, Leblois R, Whitmer A, et al Habitat continuity and geographic distance predict population genetic differentiation in giant kelp. Ecology 2010: 91: 49–56. 20380195 10.1890/09-0050.1

[25] Nanninga GB, Saenz-Agudelo P, Manica A, Berumen ML. Environmental gradients predict the genetic population structure of a coral reef fish in the Red Sea. Mol. Ecol. 2014: 23: 591–602. 10.1111/mec.12623 24320929

[26] Johansson ML, Alberto F, Reed DC, Raimondi PT, Coelho NC, Young MA, et al Seascape drivers of Macrocystis pyrifera population genetic structure in the northeast Pacific. Mol. Ecol 2015: 24: 4866–4885. 10.1111/mec.13371 26339775

[27] Kool JT, Paris CB, Andréfouët S, Cowen RK. Complex migration and the development of genetic structure in subdivided populations: an example from Caribbean coral reef ecosystems. Ecography 2010;33: 597–606.

[28] Kool JT, Paris CB, Barber PH, Cowen RK. Connectivity and the development of population genetic structure in Indo-West Pacific coral reef communities. Glob. Ecol. Biogeogr. 2011;20: 695–706.

[29] Galindo HM, Olson DB, Palumbi SR. Seascape genetics: a coupled oceanographic-genetic model predicts population structure of Caibbean corals. Current Biology 2006;16: 1622–1626. 10.1016/j.cub.2006.06.052 16920623

[30] Andréfouët S, Cabioch G, Flammand B, Pelletier B. A reappraisal of the diversity of geomorphological and genetic processes of New Caledonian coral reefs: a synthesis from optical remote sensing, coring and acoustic multibeam observations. Coral Reefs 2009;28: 691–707.

[31] Andréfouët S, Muller-Karger FE, Robinson JA, Kranenburg CJ, Torres-Pulliza D, Spraggins SA, et al. Global assessment of modern coral reef extent and diversity for regional science and management applications: a view from space. In: Proc. 10th int. Coral Reef Sym. Okinawa, 2006. pp. 1732–1745.

[32] Cravatte S, Kestenare E, Eldin G, Ganachaud A, Lefèvre J, Marin F, et al Regional circulation around New Caledonia from two decades of observations. J. Marine Syst. 2015;148: 249–271.

[33] Couvelard X, Marchesiello P, Gourdeau L, Lefèvre J. Barotropic zonal jets induced by islands in the Southwest Pacific. J. Phys. Oceanogr. 2008;38: 2185–2204.

[34] Gilbert A, Dumas P, Andréfouët S. Statut des populations des espèces de bénitiers (Tridacnidae) de Nouvelle Calédonie à travers une méta-analyse des études réalisées depuis 2004. In: Statut des populations, impacts de l’exploitation & connectivité. Nouméa: Institut de Recherche pour le Développement; 2011. 87 pp.

[35] Wantiez L, Frolla P, Goroparawa D, Keller F. Communautés biologiques et habitats coralliens des atolls d’Entrecasteaux. Etat des lieux 2012. Maintien de l’intégrité du bien. Nouméa: Université de la Nouvelle-Calédonie; 2013. 76 pp.

[36] Friedman KJ, Pakoa K, Kronen M, Chapman LB, Sauni S, Vigliola L, et al. Pacific Regional Oceanic and Coastal Fisheries Development Programme (PROCFish / C / CoFish) Vanuatu Country Report: Profiles and Results from Survey Work at Paunangisu Village, Moso Island, Uri and Uripiv Islands and the Maskelyne Archipelago. Noumea: Secretariat of the Pacific Community; 2003. 357 pp.

[37] Jameson SC. Early life history of the giant clams Tridacna crocea Lamarck, Tridacna maxima (Röding) and Hippopus hippopus (Linnaeus). Pac. Sci. 1976;30: 219–233.

[38] Lucas JS. Giant clams: description, distribution and life history In: Copland JW, Lucas JS, editors. Giant clams in Asia and the Pacific. ACIAR Monograph 9; 1988 pp 21–33.

[39] Dumas P, Tiavouane J, Senia J, Willam A, Dick L, Fauvelot C. Evidence of early chemotaxis contributing to active habitat selection by the sessile giant clam Tridacna maxima. J. Exp. Mar. Biol. Ecol. 2014;452: 63–69.

[40] Neo ML, Vicentuan K, Teo SLM, Erftemeijer PLA, Todd PA. Larval ecology of the fluted giant clam, Tridacna squamosa, and its potential effects on dispersal models. J. Exp. Mar. Biol. Ecol. 2015;469: 76–82.

[41] Wood S, Paris CB, Ridgwell A, Hendy EJ. Modelling dispersal and connectivity of broadcast spawning corals at the global scale. Glob. Ecol. Biogeogr. 2014;23: 1–11.

[42] Carr SD, Capet XJ, McWilliams JC, Pennington JT, Chavez FP. The influence of diel vertical migration on zooplankton transport and recruitment in an upwelling region: estimates from a coupled behavioral-physical model. Fish. Oceanogr. 2008;17: 1–15.

[43] Shchepetkin AF, McWilliams JC. The regional oceanic modelling system (ROMS): a split-explicit, free-surface, topography-following-coordinate oceanic model. Ocean Model. 2005;9: 347–404.

[44] Simons RD, Siegel SA, Brown KS. Model sensitivity and robustness in the estimation of larval transport: a study of particle tracking parameters. J. Mar. Sys. 2013;119–120: 19–29.

[45] Watson JR, Mitarai S, Siegel DA, Caselle JE, Dong C, McWilliams JC. Realized and potential larval connectivity in the Southern California Bight. Mar. Ecol. Progr. Ser. 2010;401: 31–48.

[46] Grulois D, Tiavouane J, Dumas PP, Fauvelot C. Isolation and characterization of fifteen microsatellite loci for the giant clam Tridacna maxima. Conserv. Genet. 2015;7: 73–75.

[47] Belkhir K, Borsa P, Chikhi L, Raufaste N, Bonhomme F. GENETIX 4.05, Population genetics software for Windows TM. Montpellier: Université de Montpellier II; 2004.

[48] Weir BS, Cockerham CC. Estimating F-statistics for the analysis of population structure. Evolution 1984;38: 1358–1370. 28563791 10.1111/j.1558-5646.1984.tb05657.x

[49] Raymond M, Rousset F. GENEPOP (version 1.2): population genetics software for exact tests and ecumenicism. Journal of heredity 1995;86: 248–249.

[50] Rice WR. Analysing tables of statistical tests. Evolution 1989;43: 223–225. 28568501 10.1111/j.1558-5646.1989.tb04220.x

[51] Chapuis MP, Estoup A. Microsatellite null alleles and estimation of population differentiation. Mol. Biol. Evol. 2007;24: 621–631. 10.1093/molbev/msl191 17150975

[52] Weir BS, Cockerham CC. Genetic Data Analysis, II Methods for discrete population genetic data. Sunderland: Sinauer Associates; 1996.

[53] Cavalli-Sforza LL, Edwards AWF. Phylogenetic analysis. Models and estimation procedures. Am. J. Hum. Genet.1967;19: 233–257. 6026583 PMC1706274

[54] Goudet J, Raymond M, De-Meeüs T, Rousset F. Testing differentiation in diploid populations. Genetics 1996;144: 1933–1940. 8978076 10.1093/genetics/144.4.1933 PMC1207740

[55] Iacchei M, Ben-Horin T, Selkoe K, Bird CE, García-Rodríguez FJ, Toonen RJ. Combined analyses of kinship and FST suggest potential drivers of chaotic genetic patchiness in high gene-flow populations. Mol. Ecol. 2013;22: 3476–94. 10.1111/mec.12341 23802550 PMC3749441

[56] Selkoe KA, Watson JR, White C, Horin TB, Iacchei M, Mitarai S, et al Taking the chaos out of genetic patchiness: seascape genetics reveals ecological and oceanographic drivers of genetic patterns in three temperate reef species. Mol. Ecol. 2010;19: 3708–3726. 10.1111/j.1365-294X.2010.04658.x 20723063

[57] Christie MR, Johnson DW, Stallings CD, Hixon MA. Self-recruitment and sweepstakes reproduction amid extensive gene flow in a coral-reef fish. Mol. Ecol. 2010;19: 1042–57. 10.1111/j.1365-294X.2010.04524.x 20089121

[58] Orsini L, Mergeay J, Vanoverbeke J, De Meester L. The role of selection in driving landscape genomic structure of the waterflea Daphnia magna. Mol. Ecol. 2013: 22: 583–601. 10.1111/mec.12117 23174029

[59] Ellner S, Hairston JNG. Role of overlapping generations in maintaining genetic variation in a fluctuating environment. Am. Nat. 1994;143: 403–417.

[60] Legendre P, Fortin MJ, Borcard D. Should the Mantel test be used in spatial analysis? Methods Ecol. Evol. 2015;6: 1239–1247.

[61] Guillot G, Rousset F. Dismantling the Mantel tests. Methods Ecol. Evol. 2013;4: 336–344.

[62] Benzie JAH, Williams ST. Genetic structure of giant clam (Tridacna maxima) populations in the West Pacific is not consistent with dispersal by present-day ocean currents. Evolution 1997;51: 768–783. 28568595 10.1111/j.1558-5646.1997.tb03660.x

[63] Kittiwattanawong K. Genetic structure of giant clam, Tridacna maxima in the Andaman Sea, Thailand. Phuket Mar. Biol. Cent. Spec. Pub. 1997;17: 109–114.

[64] DeBoer TS, Subia MD, Ambariyanto, Erdmann MV, Kovitvongsa K, Barber PH. Phylogeography and limited genetic connectivity in the endangered boring giant clam across the Coral Triangle. Conserv. Biol. 2008;22: 1255–1266. 10.1111/j.1523-1739.2008.00983.x 18637905

[65] Bonneton P, Lefebvre JP, Bretel P, Ouillon S, Douillet P. Tidal modulation of wave-setup and wave-induced currents on the Aboré coral reef, New Caledonia. J. Coastal. Res. 2007;50: 762–766.

[66] Lowe RJ, Falter JL. Oceanic forcing of coral reefs. Annu. Rev. Mar. Sci. 2015;7: 43–66. 10.1146/annurev-marine-010814-015834 25251270

[67] Cuif M, Kaplan DM, Lefèvre J, Faure VM, Caillaud M, Verley P, et al Wind- induced variability in larval retention in a coral reef system: A biophysical modelling study in the South-West Lagoon of New Caledonia. Prog. Oceanogr. 2014;122: 105–115.

[68] Selkoe KA, Henzler CM, Gaines SD. Seascape genetics and the spatial ecology of marine populations. Fish. Fish. 2008;9: 363–377.

[69] Jones GP, Russ GR, Sale PF, Steneck RS. Theme section on “Larval connectivity, resilience and the future of coral reefs”. Coral Reefs 2009;28: 303–305.

