# Supplementary material for: Considering reefscape configuration and composition in biophysical models advance seascape genetics
Source: PLoS One. 2017 May 25;12(5):e0178239. doi: 10.1371/journal.pone.0178239 (PMC5444781; doi:10.1371/journal.pone.0178239)
Supplement: S1 Table — n: number of sampled individuals; Hobs: observed heterozygosity; Hexp: Nei 's unbiased expected heterozygosity; FIS: Weir and Cockerham’s (1984) estimate of Wright’s (1951) fixation index (italic type indicate significant deviations from HWE after standard Bonferroni correction). Full names of abbreviated sampled locations are given in Fig 2. (PDF) [file pone.0178239.s001.pdf]

S1 Table. **Summary of genetic diversity at 15 microsatellite loci from *Tridacna maxima* samples**. n: number of sampled individuals; H_{obs}: observed heterozygosity; H_{exp}: Nei 's unbiased expected heterozygosity; F_{IS}: Weir and Cockerham's (1984) estimate of Wright's (1951) fixation index (italic type indicate significant deviations from HWE after standard Bonferroni correction). Full names of abbreviated sampled locations are given in Fig 2.

	AST	BB	LIF	MAR	TIG	OUV	COO	BEL	POU	VOH	BOU	GOR	CS	IP	MER	HIE	PWE	KUA	PB	CHE	SUR	HUO	EFA
**n**	**45**	**46**	**46**	**47**	**25**	**48**	**33**	**24**	**45**	**47**	**46**	**22**	**41**	**42**	**41**	**43**	**45**	**11**	**38**	**19**	**43**	**38**	**14**
**Tm14538**																							
H_E	0.879	0.864	0.888	0.892	0.856	0.887	0.907	0.851	0.850	0.889	0.903	0.855	0.867	0.889	0.886	0.880	0.865	0.818	0.893	0.843	0.872	0.890	0.829
H_O	0.714	0.545	0.630	0.680	0.560	0.553	0.727	0.608	0.488	0.587	0.695	0.590	0.625	0.642	0.650	0.609	0.534	0.636	0.763	0.526	0.571	0.621	0.642
F_{IS}	0.200	*0.379*	0.300	*0.247*	*0.364*	0.386	*0.214*	0.306	*0.434*	*0.350*	0.240	*0.330*	*0.291*	*0.288*	0.279	*0.318*	*0.392*	0.267	0.159	0.399	0.356	*0.314*	0.259
**Tm18921**																							
H_E	0.911	0.913	0.909	0.927	0.898	0.901	0.909	0.891	0.910	0.902	0.917	0.886	0.931	0.890	0.931	0.912	0.876	0.880	0.915	0.892	0.891	0.902	0.824
H_O	0.476	0.590	0.375	0.488	0.363	0.444	0.250	0.260	0.365	0.418	0.500	0.523	0.625	0.500	0.631	0.486	0.387	0.363	0.666	0.647	0.435	0.444	0.428
F_{IS}	*0.487*	*0.363*	*0.596*	0.482	*0.610*	*0.515*	*0.733*	*0.718*	*0.606*	*0.545*	*0.464*	0.429	*0.340*	*0.449*	*0.334*	*0.477*	*0.570*	*0.617*	*0.284*	0.303	*0.521*	*0.518*	*0.508*
**Tm23670**																							
H_E	0.854	0.894	0.845	0.912	0.892	0.905	0.878	0.874	0.861	0.900	0.859	0.881	0.875	0.871	0.870	0.856	0.896	0.830	0.893	0.857	0.899	0.898	0.890
H_O	0.750	0.782	0.760	0.914	0.739	0.777	0.812	0.666	0.733	0.680	0.840	0.761	0.682	0.666	0.769	0.790	0.733	0.818	0.684	0.631	0.809	0.729	0.928
F_{IS}	0.133	0.136	0.110	0.009	0.193	0.152	0.091	0.257	0.159	*0.254*	0.033	0.159	0.232	*0.247*	0.129	0.088	0.192	0.062	0.247	*0.288*	0.112	0.201	-0.00
**Tm24162**																							
H_E	0.918	0.913	0.934	0.935	0.920	0.927	0.930	0.914	0.920	0.914	0.922	0.899	0.923	0.925	0.928	0.933	0.938	0.892	0.921	0.897	0.923	0.926	0.864
H_O	0.609	0.466	0.585	0.659	0.440	0.555	0.625	0.391	0.682	0.488	0.555	0.454	0.368	0.589	0.500	0.600	0.477	0.454	0.500	0.705	0.641	0.821	0.428
F_{IS}	*0.347*	*0.498*	*0.384*	*0.306*	*0.536*	*0.411*	*0.342*	*0.587*	*0.270*	*0.474*	*0.407*	*0.512*	*0.610*	*0.374*	*0.472*	*0.368*	*0.500*	*0.526*	*0.468*	0.243	*0.317*	0.131	0.532
**Tm24224**																							
H_E	0.860	0.822	0.886	0.868	0.736	0.828	0.861	0.767	0.898	0.879	0.846	0.863	0.851	0.887	0.863	0.798	0.812	0.835	0.850	0.846	0.889	0.876	0.862
H_O	0.444	0.243	0.394	0.357	0.350	0.333	0.160	0.333	0.382	0.384	0.325	0.500	0.323	0.387	0.342	0.277	0.342	0.125	0.352	0.333	0.472	0.281	0.214
F_{IS}	*0.494*	*0.710*	*0.564*	*0.597*	*0.543*	*0.606*	*0.821*	*0.585*	*0.585*	*0.572*	*0.624*	0.442	*0.629*	*0.575*	*0.612*	*0.660*	*0.588*	*0.868*	*0.595*	*0.628*	*0.480*	*0.687*	*0.767*
**Tm2534**																							
H_E	0.659	0.572	0.656	0.677	0.653	0.618	0.680	0.544	0.669	0.677	0.737	0.740	0.649	0.644	0.690	0.739	0.561	0.615	0.704	0.681	0.750	0.702	0.606
H_O	0.238	0.225	0.189	0.219	0.272	0.257	0.275	0.181	0.297	0.289	0.414	0.150	0.333	0.181	0.459	0.305	0.088	0.200	0.058	0.312	0.135	0.333	0.307
F_{IS}	*0.646*	*0.615*	*0.719*	*0.683*	*0.598*	*0.594*	*0.606*	*0.679*	*0.566*	*0.582*	*0.448*	*0.806*	*0.498*	*0.725*	0.347	0.596	0.847	0.702	*0.919*	0.564	*0.824*	*0.535*	0.522
**TmG1**																							
H_E	0.759	0.754	0.699	0.678	0.711	0.635	0.773	0.728	0.654	0.752	0.755	0.695	0.620	0.729	0.639	0.722	0.728	0.785	0.765	0.667	0.713	0.642	0.637
H_O	0.720	0.733	0.673	0.744	0.680	0.645	0.697	0.791	0.622	0.659	0.673	0.681	0.634	0.738	0.585	0.697	0.681	0.909	0.815	0.631	0.674	0.675	0.428
F_{IS}	0.063	0.039	0.048	-0.086	0.064	-0.006	0.114	-0.066	0.060	0.134	0.118	0.043	-0.010	0.000	0.097	0.045	0.076	-0.111	-0.053	0.081	0.066	-0.039	0.361

**Tm06526**																							
H_E	0.894	0.910	0.904	0.908	0.876	0.904	0.913	0.890	0.889	0.900	0.888	0.872	0.907	0.872	0.902	0.892	0.898	0.855	0.920	0.845	0.878	0.884	0.783
H_O	0.780	0.777	0.804	0.787	0.880	0.723	0.833	0.739	0.636	0.651	0.782	0.727	0.800	0.684	0.769	0.814	0.711	0.727	0.815	0.722	0.697	0.542	0.428
F_{IS}	0.140	*0.157*	0.121	0.144	*0.016*	0.211	0.105	0.191	*0.295*	*0.288*	0.130	0.189	0.131	0.229	0.160	0.100	0.219	0.196	0.126	0.174	0.217	*0.398*	0.482
**Tm11666**																							
H_E	0.630	0.587	0.559	0.610	0.581	0.679	0.487	0.689	0.596	0.619	0.608	0.582	0.594	0.626	0.554	0.539	0.671	0.611	0.665	0.729	0.599	0.639	0.213
H_O	0.604	0.587	0.521	0.652	0.652	0.604	0.424	0.750	0.600	0.717	0.644	0.636	0.675	0.789	0.589	0.674	0.733	0.545	0.684	0.631	0.465	0.710	0.230
F_{IS}	0.053	0.013	0.078	-0.057	-0.100	0.121	0.145	-0.067	0.005	-0.148	-0.048	-0.069	-0.123	-0.247	-0.050	-0.239	-0.082	0.155	-0.014	0.161	0.235	-0.098	-0.043
**Tm20025**																							
H_E	0.654	0.586	0.622	0.602	0.722	0.637	0.622	0.572	0.658	0.573	0.547	0.630	0.613	0.668	0.555	0.634	0.700	0.417	0.606	0.590	0.573	0.391	0.775
H_O	0.477	0.268	0.371	0.250	0.500	0.282	0.260	0.142	0.550	0.285	0.307	0.500	0.305	0.394	0.307	0.487	0.425	0.363	0.411	0.473	0.425	0.250	0.714
F_{IS}	*0.281*	*0.551*	*0.415*	*0.593*	0.331	*0.564*	*0.596*	*0.761*	0.177	*0.510*	0.449	0.231	*0.512*	*0.420*	*0.456*	*0.242*	*0.404*	0.175	0.334	0.223	0.271	0.375	0.155
**Tm23637**																							
H_E	0.785	0.818	0.831	0.815	0.814	0.791	0.770	0.770	0.789	0.660	0.795	0.829	0.816	0.836	0.854	0.778	0.801	0.612	0.798	0.867	0.627	0.784	0.503
H_O	0.184	0.205	0.343	0.312	0.058	0.282	0.192	0.000	0.181	0.266	0.270	0.333	0.208	0.172	0.285	0.185	0.344	0.285	0.307	0.428	0.071	0.200	0.250
F_{IS}	*0.771*	*0.755*	*0.597*	*0.627*	*0.932*	*0.651*	*0.759*	*1.000*	*0.776*	*0.607*	*0.668*	0.626	*0.754*	*0.800*	*0.675*	*0.770*	*0.581*	0.586	*0.627*	*0.533*	*0.890*	*0.754*	0.535
**TmB12**																							
H_E	0.882	0.857	0.881	0.877	0.870	0.862	0.888	0.881	0.878	0.854	0.888	0.885	0.879	0.875	0.865	0.837	0.880	0.756	0.865	0.819	0.864	0.876	0.836
H_O	0.863	0.790	0.733	0.760	0.708	0.708	0.909	0.625	0.772	0.695	0.804	0.761	0.658	0.714	0.800	0.785	0.795	0.454	0.815	0.833	0.857	0.783	0.571
F_{IS}	0.033	0.089	0.179	0.144	0.207	0.189	-0.00	0.311	0.132	0.197	0.106	0.163	0.263	0.195	0.088	0.074	0.108	0.438	0.071	0.012	0.021	0.119	0.350
**TmE4**																							
H_E	0.926	0.940	0.919	0.923	0.926	0.936	0.919	0.916	0.927	0.918	0.930	0.905	0.934	0.934	0.939	0.937	0.930	0.907	0.928	0.936	0.936	0.922	0.882
H_O	0.704	0.600	0.577	0.630	0.541	0.723	0.709	0.541	0.511	0.574	0.644	0.619	0.682	0.512	0.561	0.666	0.545	0.888	0.526	0.526	0.650	0.594	0.357
F_{IS}	0.250	0.372	0.381	0.327	0.433	0.237	0.244	*0.427*	*0.458*	*0.384*	0.317	0.338	*0.281*	*0.462*	*0.413*	*0.300*	*0.423*	0.079	*0.444*	0.459	*0.317*	*0.368*	*0.619*
**TmE5**																							
H_E	0.890	0.898	0.891	0.890	0.899	0.908	0.900	0.845	0.888	0.896	0.896	0.881	0.883	0.897	0.884	0.901	0.869	0.863	0.869	0.862	0.903	0.876	0.900
H_O	0.909	0.956	0.931	0.851	0.880	0.833	0.909	0.913	0.911	0.851	0.760	0.909	0.925	0.785	0.756	0.907	0.755	0.909	0.789	0.500	0.883	0.789	0.857
F_{IS}	-0.010	-0.054	-0.034	0.055	0.042	0.093	0.006	-0.058	-0.014	0.061	0.162	-0.008	-0.034	0.136	0.157	0.005	0.142	-0.005	0.105	*0.444*	0.034	0.112	0.085
**TmH2**																							
H_E	0.903	0.910	0.917	0.929	0.872	0.916	0.917	0.902	0.930	0.931	0.917	0.907	0.909	0.927	0.926	0.903	0.905	0.900	0.895	0.914	0.933	0.920	0.881
H_O	0.682	0.688	0.659	0.577	0.695	0.645	0.580	0.478	0.767	0.680	0.818	0.818	0.641	0.731	0.731	0.720	0.627	0.636	0.555	0.842	0.725	0.828	0.615
F_{IS}	*0.255*	*0.254*	*0.292*	*0.388*	0.224	*0.305*	*0.381*	*0.487*	0.186	0.279	*0.120*	0.121	*0.307*	0.223	*0.222*	0.213	*0.317*	*0.336*	*0.392*	0.106	*0.235*	0.114	0.338
**Multilocus**																							
H_E	0.827	0.816	0.823	0.830	0.815	0.822	0.824	0.802	0.821	0.818	0.827	0.821	0.817	0.831	0.819	0.817	0.822	0.772	0.832	0.816	0.817	0.808	0.752
H_O	0.610	0.564	0.570	0.592	0.554	0.558	0.557	0.495	0.566	0.548	0.602	0.597	0.565	0.566	0.582	0.600	0.545	0.554	0.583	0.583	0.567	0.573	0.493
F_{IS}	*0.273*	*0.319*	*0.318*	*0.296*	*0.340*	*0.331*	*0.338*	*0.402*	*0.321*	*0.339*	*0.282*	*0.295*	*0.320*	*0.331*	*0.301*	*0.277*	*0.348*	*0.329*	*0.312*	*0.313*	*0.317*	*0.304*	*0.379*
