# Supplementary material for: Considering reefscape configuration and composition in biophysical models advance seascape genetics
Source: PLoS One. 2017 May 25;12(5):e0178239. doi: 10.1371/journal.pone.0178239 (PMC5444781; doi:10.1371/journal.pone.0178239)
Supplement: S2 Table — Above diagonal: pairwise FST; below diagonal: Cavalli-Sforza and Edwards (1967) genetic distance used for all comparisons with simulated genetic structures. Significant genotypic differentiations are indicated in italic for P-value < 0.01 and in bold for significant after sequential Bonferroni correction. Full names of abbreviated sampled locations are given in Fig 2. (PDF) [file pone.0178239.s002.pdf]

S2 Table. **Observed pairwise genetic differentiation among *T. maxima* sampled locations**. Above diagonal: pairwise F_{ST}; below diagonal: Cavalli-Sforza and Edwards (1967) genetic distance used for all comparisons with simulated genetic structures. Significant genotypic differentiations are indicated in italic for *P*-value < 0.01 and in bold for significant after sequential Bonferroni correction. Full names of abbreviated sampled locations are given in Fig 2.

	**AST**	**BB**	**LIF**	**MAR**	**TIG**	**OUV**	**COO**	**BEL**	**POU**	**VOH**	**BOU**	**GOR**	**CS**	**IP**	**MER**	**HIE**	**PWE**	**KUA**	**PB**	**CHE**	**SUR**	**HUO**	**EFA**
**AST**		0.000	0.004	0.003	0.001	0.000	0.007	0.000	0.002	0.001	0.000	0.000	0.001	0.002	0.000	0.001	0.001	0.000	0.001	0.002	0.003	0.000	***0.024***
**BB**	0.249		0.001	0.001	0.000	0.000	0.004	0.002	0.000	0.001	0.001	0.001	0.000	0.001	0.000	0.000	0.000	0.000	0.000	0.003	0.000	0.003	***0.020***
**LIF**	0.255	0.248		0.002	0.002	0.003	*0.004*	0.008	0.002	0.003	0.003	0.001	0.003	0.000	0.001	0.002	0.001	0.011	0.000	0.008	*0.006*	0.006	***0.019***
**MAR**	0.248	0.252	0.253		0.000	0.001	0.001	0.002	0.000	0.001	0.002	0.000	0.001	0.000	0.001	0.000	0.000	0.001	0.000	0.006	0.001	0.002	***0.015***
**TIG**	0.285	0.278	0.275	0.282		0.000	0.006	0.003	0.002	0.003	0.003	0.001	0.000	0.000	0.003	0.001	0.000	0.003	0.001	0.007	0.003	0.004	***0.014***
**OUV**	0.241	0.237	0.248	0.251	0.283		*0.006*	0.000	0.000	0.001	0.000	0.000	0.000	0.000	0.000	0.001	0.000	0.000	0.000	0.000	*0.001*	0.000	***0.019***
**COO**	0.281	0.283	*0.291*	0.279	0.334	*0.291*		*0.011*	0.003	*0.005*	0.004	0.002	*0.006*	*0.004*	*0.006*	*0.004*	0.004	0.006	*0.006*	0.011	*0.006*	*0.009*	***0.016***
**BEL**	0.288	0.295	0.292	0.284	0.315	0.283	*0.326*		0.004	*0.006*	0.005	0.005	0.002	0.003	0.003	0.003	0.001	0.007	0.002	0.008	0.006	0.002	***0.027***
**POU**	0.255	0.262	0.270	0.251	0.294	0.265	0.291	0.308		0.001	0.002	0.000	0.001	0.000	0.001	0.001	0.000	0.000	0.001	0.002	0.000	*0.002*	***0.020***
**VOH**	0.255	0.250	0.264	0.263	0.285	0.254	*0.294*	*0.304*	0.259		0.001	0.003	0.001	0.000	0.000	0.001	0.001	0.001	0.000	0.004	0.000	0.001	***0.020***
**BOU**	0.250	0.253	0.248	0.250	0.297	0.257	0.288	0.306	0.262	0.253		0.001	0.000	0.002	0.001	0.001	*0.000*	0.000	0.000	0.000	0.002	*0.003*	***0.024***
**GOR**	0.291	0.282	0.283	0.300	0.320	0.285	0.327	0.331	0.283	0.304	0.290		0.000	0.000	0.000	0.001	0.001	0.003	0.000	0.001	0.001	0.000	***0.021***
**CS**	0.255	0.247	0.261	0.256	0.269	0.258	*0.299*	0.299	0.264	0.254	0.246	0.306		0.000	0.000	0.002	0.000	0.003	0.000	0.001	0.001	0.000	***0.024***
**IP**	0.275	0.269	0.258	0.259	0.285	0.261	*0.309*	0.295	0.257	0.258	0.275	0.297	0.274		0.000	0.003	-0.00	0.005	0.000	0.003	0.000	0.001	***0.024***
**MER**	0.246	0.261	0.265	0.262	0.300	0.264	*0.300*	0.298	0.276	0.261	0.258	0.286	0.254	0.263		0.001	0.001	0.002	0.000	0.000	0.000	0.000	***0.023***
**HIE**	0.268	0.263	0.264	0.261	0.283	0.268	*0.292*	0.294	0.272	0.272	0.267	0.295	0.279	0.283	0.271		0.000	0.000	0.001	*0.006*	0.002	0.002	***0.022***
**PWE**	0.253	0.235	0.258	0.252	0.292	0.244	*0.288*	0.291	0.255	0.266	*0.273*	0.302	0.270	0.252	0.264	0.266		0.000	0.000	0.003	0.001	*0.001*	***0.018***
**KUA**	0.351	0.345	0.369	0.352	0.369	0.357	0.369	0.382	0.357	0.373	0.370	0.401	0.362	0.379	0.359	0.365	0.347		0.005	0.000	0.000	0.000	*0.031*
**PB**	0.271	0.271	0.262	0.259	0.300	0.261	*0.310*	0.301	0.271	0.276	0.275	0.305	0.249	0.266	0.260	0.274	0.264	0.380		0.000	0.001	0.001	***0.022***
**CHE**	0.320	0.334	0.335	0.339	0.356	0.306	0.358	0.372	0.334	0.321	0.309	0.341	0.323	0.325	0.311	*0.339*	0.324	0.412	0.317		0.000	0.003	***0.032***
**SUR**	0.261	0.259	*0.268*	0.253	0.285	*0.265*	*0.285*	0.306	0.262	0.257	0.268	0.294	0.252	0.256	0.257	0.271	0.261	0.340	0.265	0.319		*0.003*	***0.020***
**HUO**	0.252	0.277	0.271	0.282	0.319	0.275	*0.303*	0.303	*0.282*	0.273	*0.278*	0.302	0.279	0.288	0.283	0.285	0.271	0.368	0.282	0.344	*0.295*		***0.031***
**EFA**	***0.397***	***0.399***	***0.394***	***0.388***	***0.408***	***0.405***	***0.419***	***0.425***	***0.403***	***0.393***	***0.414***	***0.439***	***0.404***	***0.412***	***0.418***	***0.421***	***0.404***	*0.467*	***0.417***	***0.459***	***0.400***	***0.424***	
