# Supplementary material for: Considering reefscape configuration and composition in biophysical models advance seascape genetics
Source: PLoS One. 2017 May 25;12(5):e0178239. doi: 10.1371/journal.pone.0178239 (PMC5444781; doi:10.1371/journal.pone.0178239)

S1 Fig. Relative probability of survival for *Tridacna maxima* larvae over the competency period. Competency period considered is from 9 to 19 days, and the relative probability of survival was considered for calculation of dispersal probability in the IBOD model.

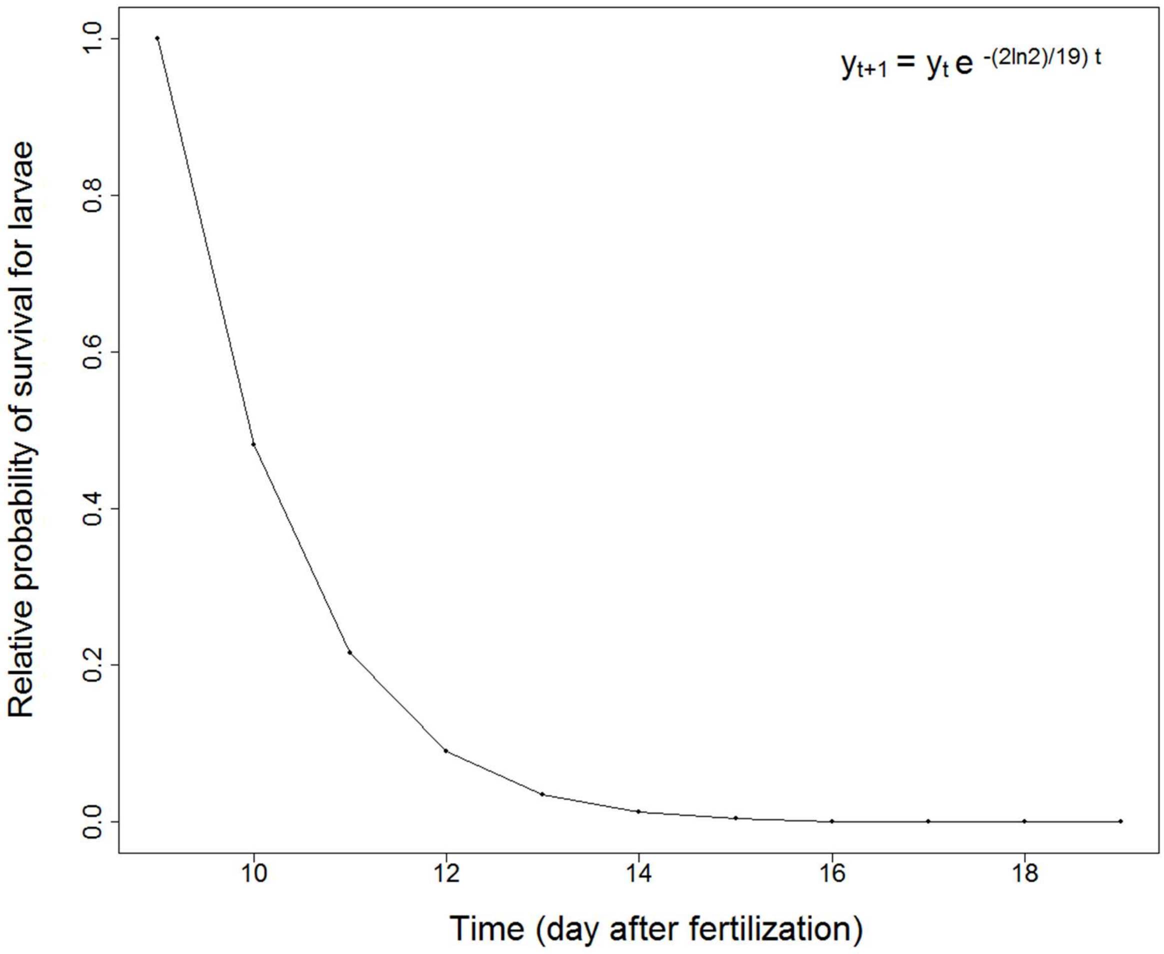

Supplement: S1 Fig — Competency period considered is from 9 to 19 days, and the relative probability of survival was considered for calculation of dispersal probability in the IBOD model. (PDF) [file pone.0178239.s003.pdf]
