# Supplementary material for: Considering reefscape configuration and composition in biophysical models advance seascape genetics
Source: PLoS One. 2017 May 25;12(5):e0178239. doi: 10.1371/journal.pone.0178239 (PMC5444781; doi:10.1371/journal.pone.0178239)

S2 Fig. Performance of IBD models to reproduce the observed genetic structure as a function of parameter *a* (see eq. 1). A) Results obtained when using the mantel coefficient of matrix correlation. B) Results obtained when using the linear R-squared. Points are medians and error bars are quantile 5% and 95%.

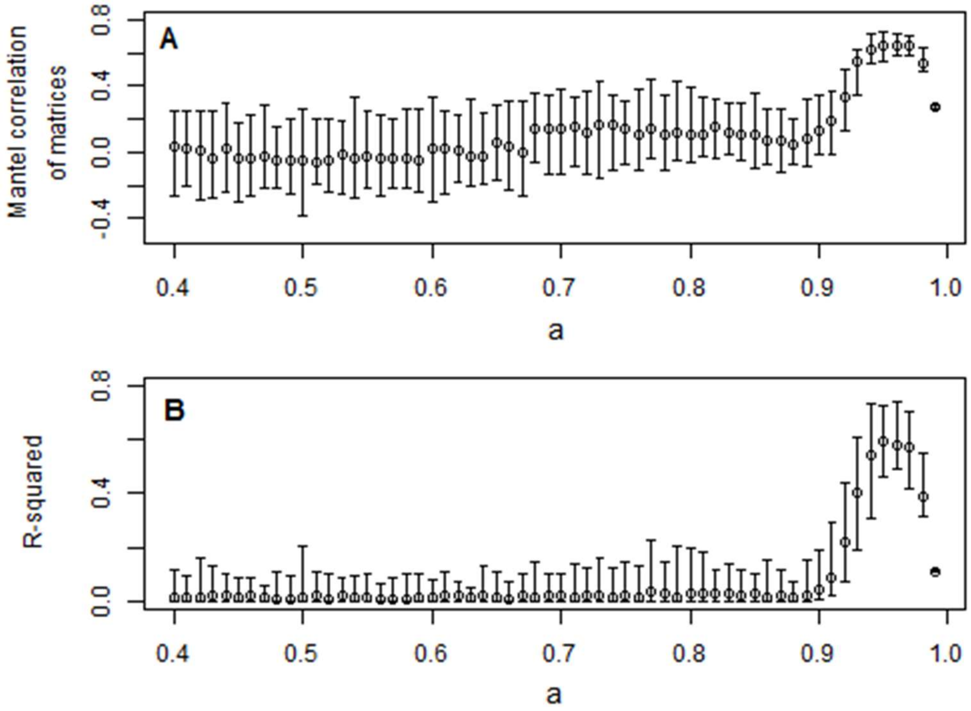

Supplement: S2 Fig — A) Results obtained when using the mantel coefficient of matrix correlation. B) Results obtained when using the linear R-squared. Points are medians and error bars are quantile 5% and 95%. (PDF) [file pone.0178239.s004.pdf]
