# Supplementary material for: Considering reefscape configuration and composition in biophysical models advance seascape genetics
Source: PLoS One. 2017 May 25;12(5):e0178239. doi: 10.1371/journal.pone.0178239 (PMC5444781; doi:10.1371/journal.pone.0178239)

S3 Fig. IBD dispersal kernel that provided best congruence between the simulated and observed genetic structures for *T. maxima* in the New Caledonia and Vanuatu area.

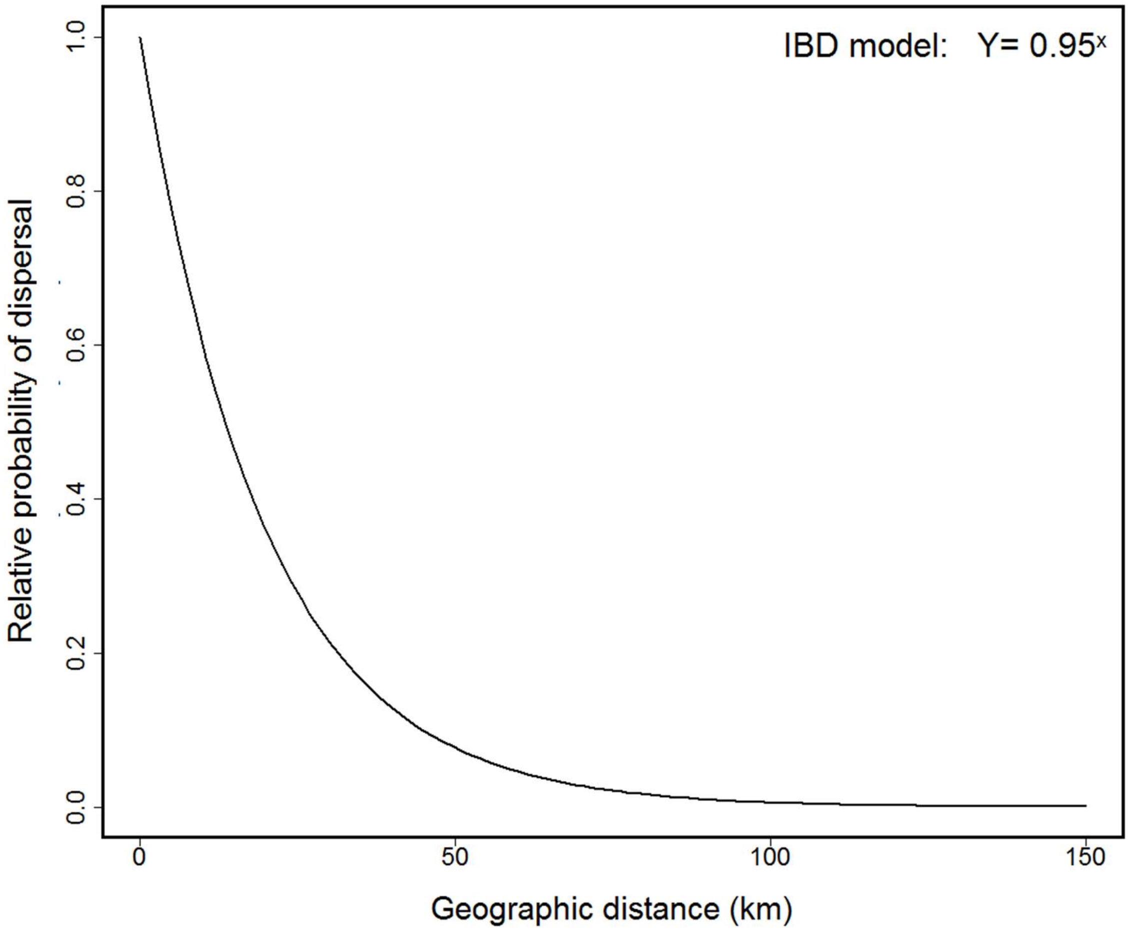

Supplement: S3 Fig — (PDF) [file pone.0178239.s005.pdf]
