# Supplementary material for: Considering reefscape configuration and composition in biophysical models advance seascape genetics
Source: PLoS One. 2017 May 25;12(5):e0178239. doi: 10.1371/journal.pone.0178239 (PMC5444781; doi:10.1371/journal.pone.0178239)
Supplement: S1 File — (PDF) [file pone.0178239.s006.pdf]

S1 File. Background surface circulation and Model configuration.

Background on the Regional Oceanic Modeling System (ROMS)

The Regional Oceanic Modeling System (ROMS; Shchepetkin and McWiliams, 2005) is the most widely used model among academic researchers and incorporates last innovations with respect to algorithmic formulation, parameterization and code implementation. The model employs the split-explicit separation of barotropic and baroclinic modes and solves the Primary Equations discretized in coastline- and terrain-following curvilinear coordinates. It is appropriated in achieving realistic multi-scale and multi-process simulations of complex flows. In the southwest Pacific, Couvelard et al. (2008) used ROMS forced with climatological mean data to evaluate model sensitivity with respect to horizontal resolution. They found the model solution at 1/12° (about 8 km) resolution suitable in reproducing mesoscale details of the mean flow deformed by both islands and the rugged bottom topography of the southwestern Pacific. A second ROMS configuration with grid resolution of 5 km but limited in duration was used in Marchesiello et al. (2010) to provide a comprehensive dynamical analysis of the coastal upwelling and heat balance around New-Caledonia during the period 2000 to 2004. They show the skills of the model to reproduce the regional circulation and the seasonal cycle of coastal upwelling after comparison against coastal observation of real upwelling events using in-situ and satellite data. Here, a ROMS simulation at 1/12° is performed over the period 1993 to 2010, a period long enough to capture main features and variability of the regional circulation

Model configuration

The regional domain is centered on New-Caledonia with a regular grid extending from 30°S to 12°S in latitude and from 153°E to 174°E in longitude. Horizontal grid resolution is 1/12° and is appropriate with regards to convergence experiments performed in Couvelard et al. (2008). There are 35 vertical levels with refinement toward the surface so that the thickness of the surface layer ranges from 0.16 to 4 m. The maximum thickness of the bottom layer is 720 m. The model bottom topography is interpolated from a 2‘ resolution database ETOPO2 from NOAA-NGDC augmented by soundings from the New Caledonia Exclusive Economic Zone (EEZ) bathymetry (BDBNC-DTSI 2009), which includes details of the geomorphology of Loyalty Islands and New Caledonia Mainland for coastal slope, shallow waters and straits.

The model grid, surface and lateral forcing conditions are built using the ROMSTOOLS package (Penven 2003).

The domain is embedded in the multi-annual OFAM global ocean model with resolution coarser than 1° but with refinement towards the Australia EEZ between 90E and 180°E in Longitude (Oke et al., 2012). More precisely, in the region, daily ocean fields from OFAM are available on a 1/10° resolution grid in the period 1993 to 2012. It is worth noting that the bottom topography in OFAM derives from the GEBCO database (Oke et al., 2012) and misses some aforementioned details of the geomorphology of New Caledonia. The ROMS solution at the four lateral boundaries is nudged toward 5-days averaged outputs of the OFAM model using Orlanski-type radiation open boundary conditions detailed in Marchesiello et al. (2001). At the surface, air-sea fluxes of momentum and sensible, latent and longwave heat are computed by applying bulk formulaes from Fairall et al. (2003) to 3-hourly meteorological fields from the interim reanalysis solution (ERA interim, Dee and Uppala, 2009) distributed by the European Centre for Medium-Range Weather Forecasts (ECMWF). The meteorological fields are also consistent with those used to force the OFAM solution. Inter-annual signals such as El Niño–Southern Oscillation (ENSO) are introduced in the model as remote forcing through the lateral boundaries using the global ocean model OFAM and as local forcing through surface fluxes, principally mediated by local surface winds (Delcroix and Lenormand, 1997). The model is initialized using ocean fields from OFAM3 in early January 1993 and integrated for two seasonal cycles using repeated surface and lateral boundaries conditions for the year 1993 (spin-up of 2 years). Then integration is continued for the period January 1993 to December 2010.

The model is initialized using ocean fields from OFAM3 in early January 1993 and integrated for two seasonal cycles using repeated surface and lateral boundaries conditions for the year 1993 (spin-up of 2 years). Then integration is continued for the period January 1993 to December 2010.

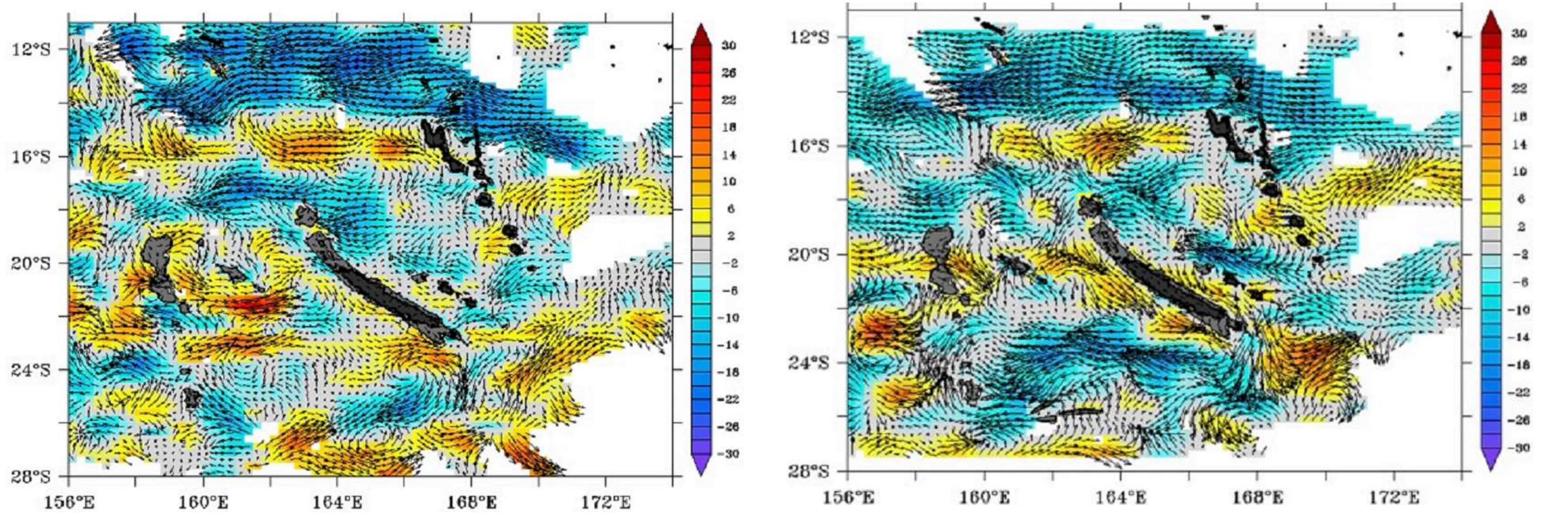

Fig. 1 Comparison of observed versus simulated (ROMS) direction and speed of currents around New Caledonia and Vanuatu. Left: Observed surface circulation (25-200m) based on 20 years observations of Shipboard Acoustic Doppler Current Profiler (SDACP) after reprocessing as detailed in Cravatte et al. (2015). Right: Mean simulated ROMS currents in the same layer and subsampled at the same locations and times as the SADCP data (Source Cravatte S.). The color bar indicates the magnitude of zonal currents (cm/s).

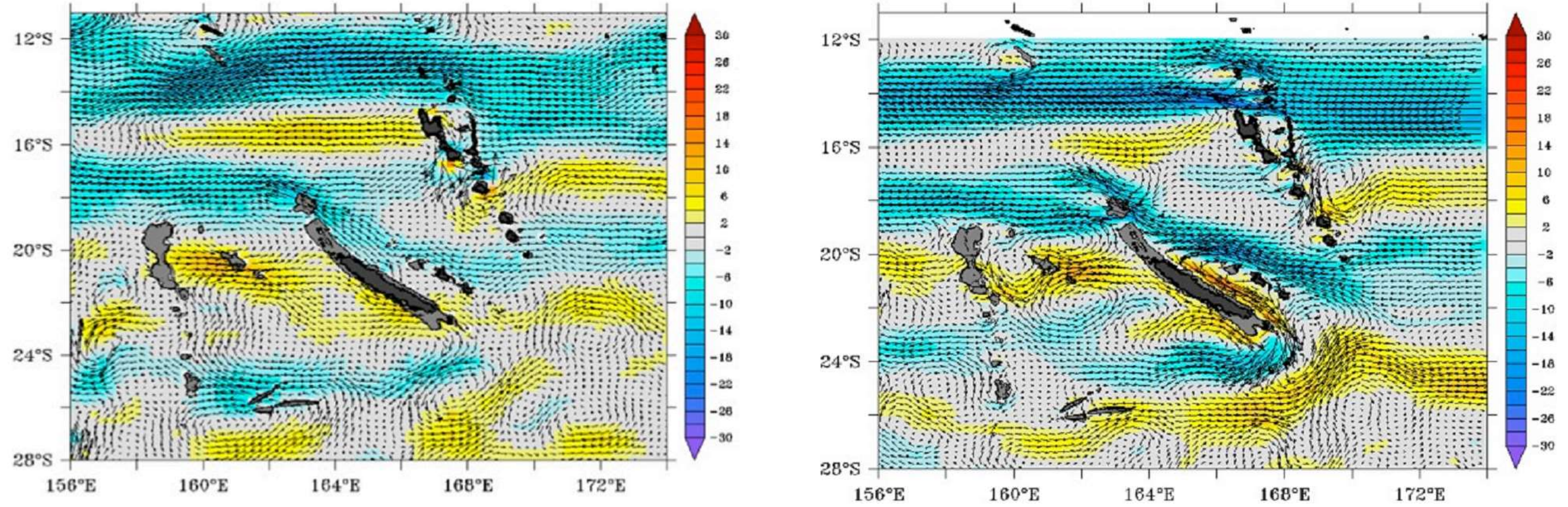

Fig. 2: Comparison of observed versus simulated (ROMS) mean surface geostrophic circulation. Left: Observed mean surface geostrophic circulation (25-100m) based on climatological hydrographic data merged with drift trajectories of Argo floats (see details in Cravatte et al. (2015)). Right: Mean simulated ROMS currents in the same layer (Source Cracatte S.). The color bar indicates the magnitude of zonal currents (cm/s).

References:

Couvelard, X., Marchesiello, P., Gourdeau, L. & Lefèvre, J. (2008). Barotropic Zonal Jets Induced by Islands in the Southwest Pacific. *J. Phys. Oceanogr.*, 38, 2185–2204.

Cravatte, S., Kestenare, E., Eldin, G., Ganachaud, A., Lefèvre J., Frédéric, *et al.* (2015) Regional circulation around New Caledonia from two decades of observations. *Journal of Marine Systems*, 2015, 148, 249-271.

Dee, D.P. & Uppala, S., (2009). Variational bias correction of satellite radiance data in the ERA interim reanalysis. *Quarterly Journal of the Royal Meteorological Society*, 135,1830-1841.

Delcroix, T. & Lenormand, O., (1997). ENSO signals in the vicinity of New Caledonia, south-western Pacicic. *O. acta* 20, 481-491.

DTSI-BDBNC (2009) : Direction des Technologies et des Services de l'Information. (2009). La Base de Données Bathymétriques de la Nouvelle-Calédonie : une contribution du gouvernement de la Nouvelle-Calédonie au programme ZoNéCo. http://sig-public.gouv.nc/notice_banque_de_donnees_bathymetriques.pdf

Fairall, C.W., Bradley, E.F., Hare, J.E., Grachev, A.A. & Edson, J. (2003). Bulk parameterization of air–sea fluxes: Updates and verification for the COARE algorithm. *J. Climate*, 16, 571–591.

Marchesiello P., McWilliams, J.C. & A. Shchepetkin, A. (2001). Open boundary condition for long-term integration of regional oceanic models. *Ocean Modelling*, 3, 1-21.

Marchesiello, P., Lefèvre J., Vega A., Couvelard X. & Menkès C., (2010) Coastal upwelling, circulation and heat balance around New Caledonia's barrier reef. In : *New Caledonia tropical lagoons: an overview of multidisciplinary investigations*. *Marine Pollution Bulletin*, 61, 432-448.

Oke, P.R., Sakov, P., Cahill, M.L., Dunn, J.R., Fiedler, R., Griffin, D.A., *et al.* (2012): Towards a dynamically balanced eddy-resolving ocean reanalysis: BRAN3, *Ocean Modelling*, 67, 52-70.

Penven, P. (2003). ROMSTOOLS user's guide (http://fraise.univ-brest.fr/penven/roms_tools/). IRD Technical Report.

Shchepetkin, A.F. & McWilliams J.C. (2005) Regional Ocean Model System: a split-explicit ocean model with a free-surface and topography-following vertical coordinate, *Ocean Modell*. 9, 347-404.
