# Supplementary material for: Considering reefscape configuration and composition in biophysical models advance seascape genetics
Source: PLoS One. 2017 May 25;12(5):e0178239. doi: 10.1371/journal.pone.0178239 (PMC5444781; doi:10.1371/journal.pone.0178239)
Supplement: S2 File — (PDF) [file pone.0178239.s007.pdf]

S2 File. Linear regression models between genetic distance, geographic distance, oceanographic distance, and habitat continuity.

This supplementary information complement the simulation approach implemented in the main article, to test with simple linear regressions if accounting for habitat continuity (HC) improve our ability to predict genetic distance (GD) compared to isolation by distance (IBD) and isolation by oceanographic distance (IBOD) models. The shortest path between each pair of sites without crossing land was identified using the GIS software QGIS2.14. This path was used to calculate the geographical distance (GeoDist) and the habitat continuity (HC) between each pair of sites. Habitat continuity was calculated as the proportion of reef habitat included in a 0.5° buffer around the shortest path between the two sites. The oceanographic distance (OceaDist) was calculated as the probability for propagules to travel from one site to another through oceanic currents (see main text). We used a linear model (lm function of package stat in R.3.3.3.) to test if adding HC as explaining variable improved the fit of GD compared to models which consider GeoDist and OceaDist alone as explaining variables. Models were compared with the Akaike Information Criterion (AIC).
The relationships between GD and GeoDist, OceaDist and HC are shown in Figure 1. Results from the linear modelling approach show that (i) GeoDist performed better than OceaDist in explaining GD; and (ii) taking HC into account significantly decreased AIC (i.e. improved models) for both IBD and IBOD models (Table 1). These results are congruent with those obtained by the simulation approach implemented in the main article.

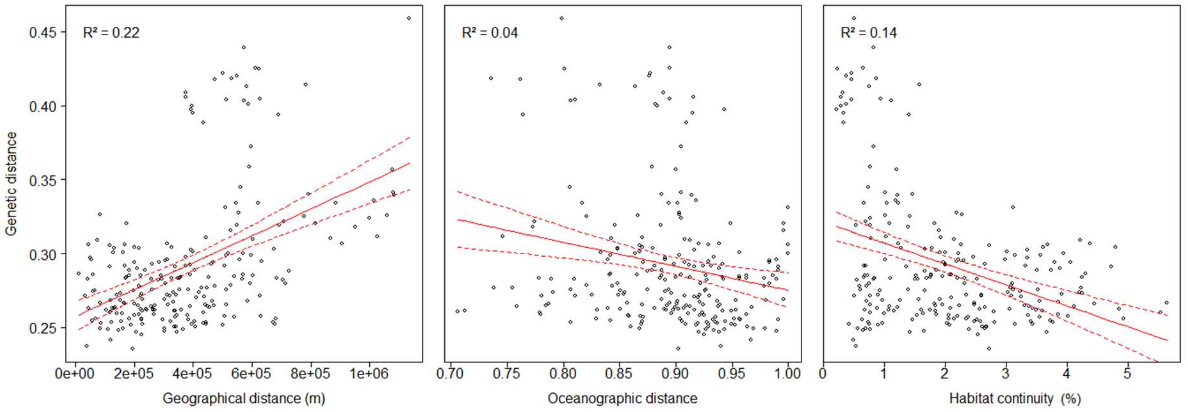

Figure 1: linear relationships between genetic distance (GD) and geographic distance (GeoDist), oceanographic distance (OceaDist), and habitat continuity (HC).

Table 1: Results from the linear regressions between GD and GeoDist and OceaDist with *versus* without taking HC into account. Significance of terms is displayed for p < 0.001 (***), p < 0.01 (**), p < 0.05 (*), and 0.05 < p 0.06 (•).

Model	Adjusted R-squared	Significance of terms	AIC
GD ~ GeoDist	0.22	GeoDist ***	-820.8455
GD ~ GeoDist x HC	0.36	GeoDist *** ; HC• ; GeoDist:HC***	-863.9063
GD ~ OceaDist	0.04	IBOD**	-773.8525
GD ~ OceaDist x HC	0.33	OceaDist*** ; HC*** ; OceaDist:HC***	-855.0713
