# Supplementary material for: Considering reefscape configuration and composition in biophysical models advance seascape genetics
Source: PLoS One. 2017 May 25;12(5):e0178239. doi: 10.1371/journal.pone.0178239 (PMC5444781; doi:10.1371/journal.pone.0178239)
Supplement: S3 File — (PDF) [file pone.0178239.s008.pdf]

S3 File. F_{st} values per locus obtained with and without using ENA.

Locus	All samples		All samples but Vanuatu	
	Not using ENA	Using ENA	Not using ENA	Using ENA
All	Mean: 0.0024<br>95%CI-low: 0.0014<br>95%CI-up: 0.0037	Mean: 0.0025<br>95%CI-low: 0.0014<br>95%CI-up: 0.0038	Mean: 0.00157<br>95%CI-low: 0.0008<br>95%CI-up: 0.0024	Mean: 0.00177<br>95%CI-low: 0.001035<br>95%CI-up: 0.002620
1	0.0001	-0.0001	-0.0005	-0.0009
2	0.0035	0.0020	0.0028	0.0015
3	0.0019	0.0028	0.0016	0.0024
4	0.0010	0.0016	0.0003	0.0008
5	0.0029	0.0020	0.0030	0.0021
6	0.0041	0.0043	0.0044	0.0042
7	-0.0018	-0.0004	-0.0015	-0.0000
8	0.0038	0.0034	0.0029	0.0029
9	0.0080	0.0064	0.0034	0.0041
10	0.0057	0.0104	0.0001	0.0059

11	0.0070	0.0042	0.0054	0.0021
12	0.0027	0.0019	0.0020	0.0015
13	0.0004	0.0011	0.0005	0.0014
14	0.0015	0.0017	0.0008	0.0009
15	0.0002	0.0003	0.0002	0.0003
