# Supplementary material for: Considering reefscape configuration and composition in biophysical models advance seascape genetics
Source: PLoS One. 2017 May 25;12(5):e0178239. doi: 10.1371/journal.pone.0178239 (PMC5444781; doi:10.1371/journal.pone.0178239)
Supplement: S4 File — (PDF) [file pone.0178239.s009.pdf]

S4 File. Indirect estimates of gene dispersal distance from empirical genetic data using Moran's *I* relationship coefficients.

To obtain indirect estimates of gene dispersal distance parameters (e.g. neighborhood size) in the context of isolation-by-distance processes (Hardy & Vekemans 1999), Moran's *I* relationship coefficients were estimated for each pair of individual within geographical distance classes using the program SPAGEDI 3.0 (Hardy & Vekemans 2002). As the number of geographical distance classes may influence results, we tested 20 classes, 15 classes, 10 classes and 5 classes.

The significance of relatedness among individuals in each geographic distance class was obtained by comparing the observed coefficient to the distribution of the statistic under the null hypothesis of no spatial structure generated using 10 000 resamplings of the data, permuting spatial location among distance groups. Such spatial autocorrelation methods have the advantage of allowing inference of spatial genetic structure independently from the often violated assumptions of classic *F*-statistics, such as absence of selection and mutation or complete random migration of a constant number of individuals between the subpopulations (Hardy & Vekemans 1999). Significant positive autocorrelation implies that individuals within a particular distance class are more genetically similar than individuals randomly taken from any distance class. Therefore, the first *x*-intercept of the correlogram, which gives the average distance at which the similarity of any 2 sites is equal to the region-wide similarity expected by chance alone, is termed the 'genetic patch size' (Sokal & Wartenberg 1983).

When 20 geographical distance classes were considered, significantly positive Moran's *I* relationship coefficients was detected between individuals within sampled sites (0 m class) I = 0.0016, $p < 0.001$), indicating that neighboring individuals had a higher genetic relatedness

than random pairs of individuals issued from all samples. The autocorrelogram (Fig. 1A) suggested an estimated patch size for *Tridacna maxima* of <100 km, as shown by the negative, non-significant values of Moran's *I* coefficient in the 100 km distance class. This result was sensitive to the number of geographical distance classes, since defining 15, 10 or 5 classes provided estimated genetic patch size > 200 km. We conclude that the Moran's *I* relationship roughly suggests genetic patch sizes between 100 and 250 km.

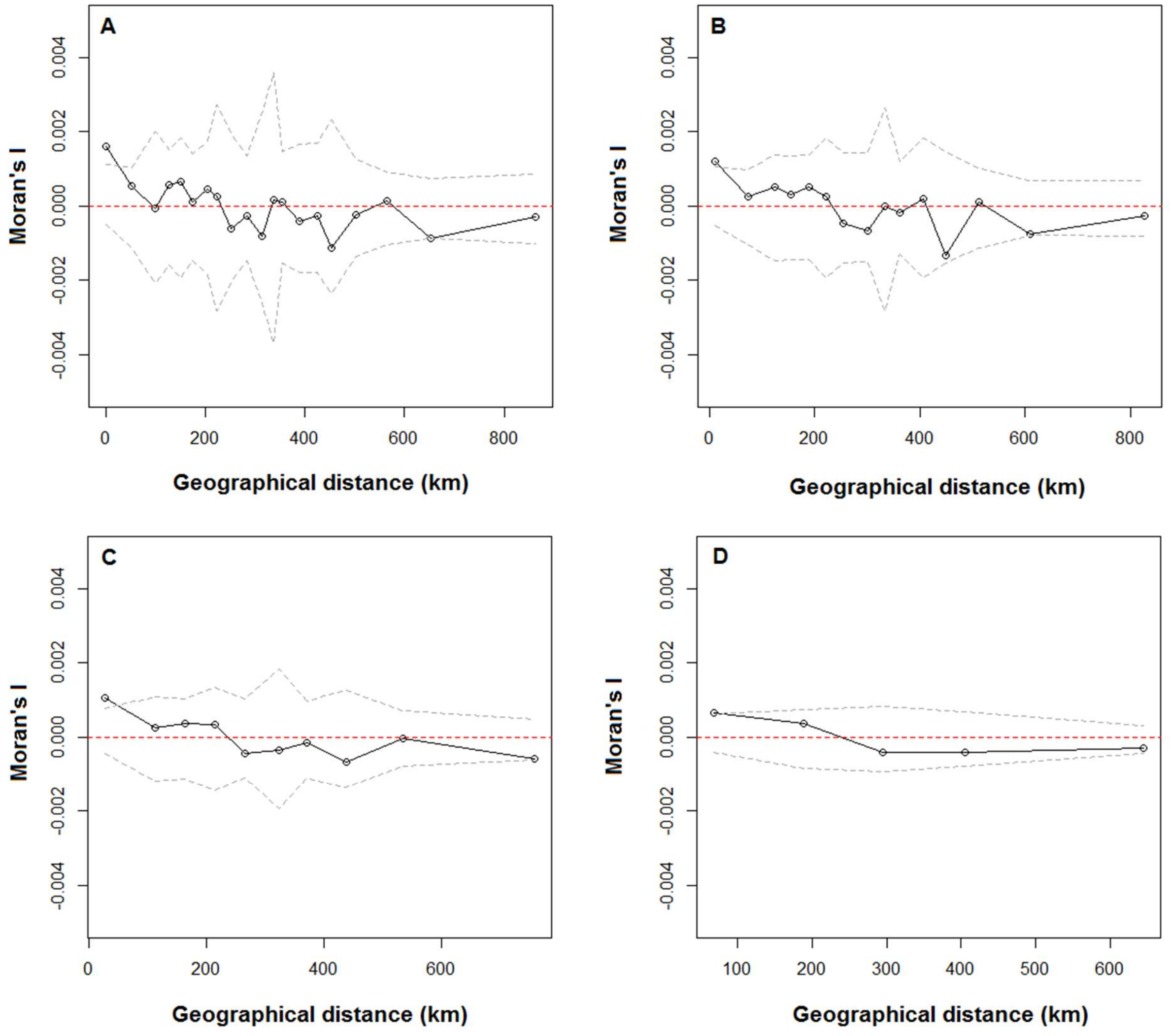

Fig. 1: Moran's I coefficient obtained when defining 20 geographical distance classes (A), 15 classes (B), 10 classes (C), and 5 classes (D).
